# Functional specialization of UDP‐glycosyltransferase 73P12 in licorice to produce a sweet triterpenoid saponin, glycyrrhizin

**DOI:** 10.1111/tpj.14409

**Published:** 2019-06-26

**Authors:** Yuhta Nomura, Hikaru Seki, Tomonori Suzuki, Kiyoshi Ohyama, Masaharu Mizutani, Tomomi Kaku, Keita Tamura, Eiichiro Ono, Manabu Horikawa, Hiroshi Sudo, Hiroaki Hayashi, Kazuki Saito, Toshiya Muranaka

**Affiliations:** ^1^ Department of Biotechnology Graduate School of Engineering Osaka University 2‐1 Yamadaoka Suita Osaka 565‐0871 Japan; ^2^ RIKEN Center for Sustainable Resource Science 1‐7‐22 Suehiro‐cho, Tsurumi‐ku Yokohama Kanagawa 230‐0045 Japan; ^3^ Department of Chemistry and Materials Science Tokyo Institute of Technology 2‐12‐1 O‐okayama, Meguro‐ku Tokyo 152‐8551 Japan; ^4^ Graduate School of Agricultural Science Kobe University 1‐1 Rokkodai‐cho, Nada‐ku Kobe Hyogo 657‐8501 Japan; ^5^ Suntory Global Innovation Center Ltd Research Institute 8‐1‐1 Seikadai, Seika‐cho, Soraku‐gun Kyoto 619‐0284 Japan; ^6^ Suntory Foundation for Life Sciences Bioorganic Research Institute 8‐1‐1 Seikadai, Seika‐cho, Soraku‐gun Kyoto 619‐0284 Japan; ^7^ Tokiwa Phytochemical Co., Ltd 158 Kinoko Sakura Chiba 285‐0801 Japan; ^8^ Graduate School of Pharmaceutical Sciences Chiba University 1‐8‐1 Inohana, Chuo‐ku Chiba 260‐8675 Japan; ^9^ School of Pharmacy Iwate Medical University 2‐1‐1 Nishitokuta Yahaba, Iwate 028‐3694 Japan; ^10^ Present address: RIKEN Center for Sustainable Resource Science 2‐1 Hirosawa Wako Saitama 351‐0198 Japan

**Keywords:** UDP‐glycosyltransferase, triterpenoid saponin, UDP‐glucuronic acid, glycyrrhizin, natural variant, functional specialization, divergent evolution, licorice, *Glycyrrhiza uralensis*

## Abstract

Glycyrrhizin, a sweet triterpenoid saponin found in the roots and stolons of Glycyrrhiza species (licorice), is an important active ingredient in traditional herbal medicine. We previously identified two cytochrome P450 monooxygenases, CYP88D6 and CYP72A154, that produce an aglycone of glycyrrhizin, glycyrrhetinic acid, in *Glycyrrhiza uralensis*. The sugar moiety of glycyrrhizin, which is composed of two glucuronic acids, makes it sweet and reduces its side‐effects. Here, we report that UDP‐glycosyltransferase (UGT) 73P12 catalyzes the second glucuronosylation as the final step of glycyrrhizin biosynthesis in *G*. *uralensis*; the UGT73P12 produced glycyrrhizin by transferring a glucuronosyl moiety of UDP‐glucuronic acid to glycyrrhetinic acid 3‐*O*‐monoglucuronide. We also obtained a natural variant of UGT73P12 from a glycyrrhizin‐deficient (83‐555) strain of *G*. *uralensis*. The natural variant showed loss of specificity for UDP‐glucuronic acid and resulted in the production of an alternative saponin, glucoglycyrrhizin. These results are consistent with the chemical phenotype of the 83‐555 strain, and suggest the contribution of UGT73P12 to glycyrrhizin biosynthesis *in planta*. Furthermore, we identified Arg32 as the essential residue of UGT73P12 that provides high specificity for UDP‐glucuronic acid. These results strongly suggest the existence of an electrostatic interaction between the positively charged Arg32 and the negatively charged carboxy group of UDP‐glucuronic acid. The functional arginine residue and resultant specificity for UDP‐glucuronic acid are unique to UGT73P12 in the UGT73P subfamily. Our findings demonstrate the functional specialization of UGT73P12 for glycyrrhizin biosynthesis during divergent evolution, and provide mechanistic insights into UDP‐sugar selectivity for the rational engineering of sweet triterpenoid saponins.

## Significance Statement

Glycyrrhizin, a licorice‐derived sweet triterpenoid diglycoside, is one of the most important phytoconstituents in the pharmaceutical and food industries. Here, we identified UGT73P12 as the enzyme catalyzing the sugar–sugar linkage to produce glycyrrhizin from its less glycosylated precursor in the presence of UDP‐glucuronic acid, and found that Arg32 of UGT73P12 is essential for the high specificity for UDP‐glucuronic acid, which was probably acquired during evolution to enable the functional specialization of UGT73P12 for glycyrrhizin biosynthesis.

## Introduction

Glycyrrhiza species are medicinally important leguminous plants, and their thickened main roots and stolons (licorice) have been used extensively in traditional herbal medicine in China for thousands of years. The primary active ingredient found only in licorice is glycyrrhizin, which is an oleanane‐type triterpenoid saponin that has many pharmacological effects, such as anti‐inflammatory, anti‐cancer and hepatoprotective activities (Zhang and Ye, 2009; Wang *et al*., 2013; Kao *et al*., 2014). Glycyrrhizin is also a natural non‐caloric sweetener that is 150 times sweeter than sucrose and has been used as a food additive (Kitagawa, 2002; DuBois and Prakash, 2012).

**Figure 1:**
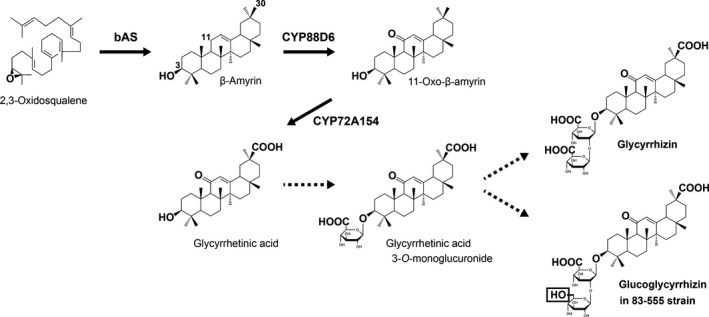
Proposed biosynthetic pathways for glycyrrhizin and glucoglycyrrhizin in *Glycyrrhiza uralensis*. The following terms are used: bAS, β‐amyrin synthase; CYP88D6, β‐amyrin 11‐oxidase; and CYP72A154, 11‐oxo‐β‐amyrin 30‐oxidase. The positions of carbon atoms related to these pathways are indicated on the chemical structure of β‐amyrin. Broken arrows indicate unidentified and expected routes to produce glycyrrhizin and glucoglycyrrhizin in the glycyrrhizin‐producing and ‐deficient (83‐555) strains, respectively.

The biosynthesis of glycyrrhizin involves an initial origami‐like cyclization of 2,3‐oxidosqualene to produce the common skeleton of oleanane‐type triterpenoids, β‐amyrin (Figure 1). This reaction is catalyzed by the oxidosqualene cyclase β‐amyrin synthase (Hayashi *et al*., 2001; Augustin *et al*., 2011; Thimmappa *et al*., 2014). This reaction is followed by site‐specific oxidation of β‐amyrin at the C‐11 and C‐30 positions, which is catalyzed by two cytochrome P450 monooxygenases, CYP88D6 (β‐amyrin 11‐oxidase) and CYP72A154 (11‐oxo‐β‐amyrin 30‐oxidase), respectively (Seki *et al*., 2008, 2011). The oxidized form of β‐amyrin, 30‐carboxy‐11‐oxo‐β‐amyrin, is generally designated as glycyrrhetinic acid and corresponds to an aglycone of glycyrrhizin (Figure 1). The sugar moiety of glycyrrhizin, which is composed of two glucuronic acids, makes it sweet and reduces its side‐effects, such as apparent mineralocorticoid excess syndrome after oral administration (Sontia *et al*., 2008; Omar *et al*., 2012; Kao *et al*., 2014; Mizutani *et al*., 2014). This sugar moiety is expected to be added through a two‐step glucuronosylation of glycyrrhetinic acid at the C‐3 hydroxy group by glucuronosyltransferases, resulting in the sequential production of glycyrrhetinic acid 3‐*O*‐monoglucuronide and glycyrrhizin (Figure 1).

The most plausible candidate glucuronosyltransferases are family 1 glycosyltransferases, usually referred to as uridine diphosphate (UDP)‐glycosyltransferases (UGTs; Augustin *et al*., 2011; Thimmappa *et al*., 2014; Seki *et al*., 2015). Plant UGTs are generally known as a group of enzymes that transfer a sugar moiety of UDP‐sugars to specific natural products, including plant secondary metabolites such as triterpenoids, steroids and phenylpropanoids, in a regiospecific manner (Bowles *et al*., 2006). However, how the specificity of UDP‐sugar donors and acceptor substrates is controlled in UGTs is poorly understood. It is especially hard to predict the UDP‐sugar selectivity from the primary sequence (Yonekura‐Sakakibara and Hanada, 2011; Caputi *et al*., 2012). UDP‐glucose is most commonly used by plant UGTs, but others, including UDP‐galactose, UDP‐arabinose, UDP‐rhamnose, UDP‐xylose and UDP‐glucuronic acid, are also utilized by specialized UGTs (Bowles *et al*., 2006). Among these UDP‐sugars, only UDP‐glucuronic acid has a negatively charged carboxy group in its sugar moiety. Thus, there is expected to be an additional molecular mechanism stabilizing the negative charge in the catalytic site of all glucuronosyltransferases to confer a high affinity for UDP‐glucuronic acid, as supported by previous studies on flavonoid‐related UGTs (Osmani *et al*., 2008; Noguchi *et al*., 2009; Ono *et al*., 2010).

A previous comparative analysis of various strains of *Glycyrrhiza uralensis* revealed the presence of a glycyrrhizin‐deficient strain 83‐555, in which glucoglycyrrhizin was predominantly accumulated (Hayashi *et al*., 2013). Glucoglycyrrhizin shares a common structure to glycyrrhetinic acid 3‐*O*‐monoglucuronide with glycyrrhizin, but possesses a glucose as an alternative second sugar attached to glycyrrhetinic acid 3‐*O*‐monoglucuronide (Figure 1). This chemical phenotype strongly suggests that the 83‐555 strain harbors mutations in a UGT gene responsible for the glucuronosylation of glycyrrhetinic acid 3‐*O*‐monoglucuronide, leading to a change in its sugar donor specificity from UDP‐glucuronic acid to UDP‐glucose. Therefore, the 83‐555 strain is helpful not only to evaluate the correlation between the production of glucoglycyrrhizin *in planta* and the sugar donor specificity of the UGT protein, but also to identify important residues of the UGT protein to provide the high specificity for UDP‐glucuronic acid.

We previously performed a transcriptome analysis of *G. uralensis* with RNA sequencing (RNA‐Seq; Ramilowski *et al*., 2013). In the present study, we further examined the transcriptome data and successfully identified a UGT73P12 protein that produces glycyrrhizin by transferring a glucuronosyl moiety of UDP‐glucuronic acid to glycyrrhetinic acid 3‐*O*‐monoglucuronide. We then confirmed that a natural variant of UGT73P12 obtained from the 83‐555 strain preferentially selects UDP‐glucose as a sugar donor and produces glucoglycyrrhizin as the alternative product, in accordance with the chemical phenotype of the 83‐555 strain. In addition, we identified Arg32 as the essential residue of UGT73P12 protein to provide the high specificity for UDP‐glucuronic acid using protein structure modeling and residue‐swapping experiments. Finally, we present a reasonable model of the molecular mechanism controlling the sugar donor specificity of UGT73P12 protein.

## Results

### Transcriptome data mining for candidate UGTs involved in glycyrrhizin biosynthesis

The biosynthesis of glycyrrhizin in licorice plants occurs in their underground organs (thickened main roots and stolons), but not in their aerial parts (Hayashi *et al*., 1993). In addition, it is known that the active growth of the aerial parts is needed to produce glycyrrhizin in the thickened main roots (Hayashi *et al*., 2004). Thus, the productivity of glycyrrhizin in licorice plants depends on the season and furthermore varies among their strains (Hayashi *et al*., 2005). On the basis of these observations, we previously performed a transcriptome analysis of *G. uralensis* with RNA‐Seq of four cDNA libraries that were obtained from four different samples as follows: Library 1 consisted of roots of the high‐glycyrrhizin‐producing strain 308‐19 harvested in June, when the aerial parts were growing; Library 2 consisted of roots of the 308‐19 strain harvested in December, when the aerial parts were dormant; Library 3 consisted of roots of the low‐glycyrrhizin‐producing strain 87‐458 harvested in June; and Library 4 consisted of leaves of the 308‐19 strain harvested in June (Ramilowski *et al*., 2013). This analysis provided 43 882 unigene sequences and their expression profiles in each of the four libraries.

In this study, we further examined the transcriptome data to extract candidate UGTs involved in glycyrrhizin biosynthesis from the unigene sequences. It is generally known that plant UGTs possess a highly conserved 44‐amino‐acid sequence, designated as a plant secondary product glycosyltransferase (PSPG) motif, in their C‐terminal region to provide a binding site for a UDP moiety of UDP‐sugars, whereas the other region shows relatively low similarity in the primary sequence (Yonekura‐Sakakibara and Hanada, 2011; Caputi *et al*., 2012). Therefore, a dataset of amino acid sequences containing the PSPG motif, which is available in the Pfam database (Sonnhammer *et al*., 1998) as a hidden Markov model‐based profile of the UGT domain (Pfam family: PF00201), is commonly used as a query to find plant UGTs in translated nucleotide databases. We first searched the unigene sequences for the possible open reading frames encoding the UGT domain and annotated 90 unigenes as putative UGTs. A recent report on the draft genome assembly of *G*.* uralensis* revealed the presence of 91 UGTs in *G*.* uralensis* (Mochida *et al*., 2017), indicating the high coverage of our dataset of unigene sequences.

**Figure 2:**
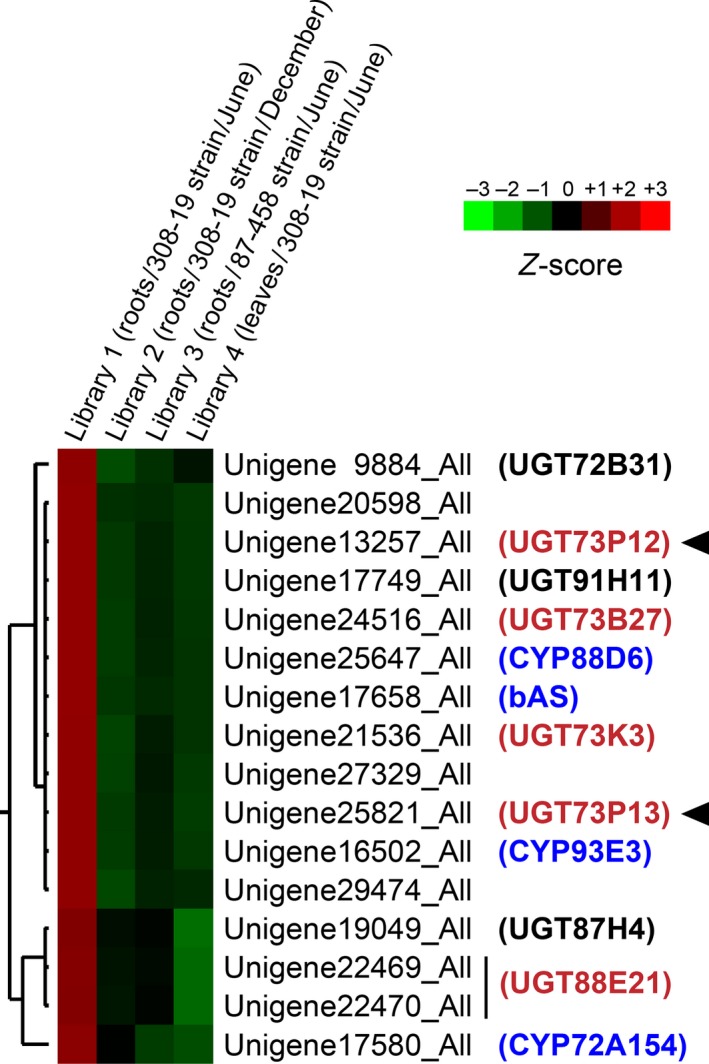
Hierarchical clustering of unigene expression profiles in *Glycyrrhiza uralensis*. The heatmap shows the relative expression level of unigenes in four libraries. The color scale (−3.00 to 3.00 from green to red, respectively) represents the *Z*‐score, which corresponds to the relative expression level. Only the expanded heatmap is shown here to show the unigenes with expression patterns highly correlated with those of other glycyrrhizin and/or soyasaponin biosynthetic genes, *bAS*,* CYP88D6*,* CYP72A154* and *CYP93E3* (blue). The complete heatmap is shown in Figure S1. The bar spanning two unigenes indicates that these unigenes are derived from one gene. The dendrogram along the left side of heat maps indicates the hierarchical clustering of unigenes. The UGTs shown in black or red were named in this study, and those shown in red were further used to screen for functional proteins. Arrowheads indicate the UGTs characterized in this study.

To refine the candidate UGTs further, we next checked their expression profiles in each of the four libraries. The expression levels were previously calculated in fragments per kilobase per million reads (FPKM) units. We normalized the FPKM values as *Z*‐score values among the four libraries, and performed hierarchical clustering of the *Z*‐score values. The results revealed that 12 of the 90 unigenes showed highly correlated expression patterns with the previously identified glycyrrhizin biosynthetic genes *bAS*,* CYP88D6* and *CYP72A154* across the four different libraries (Figures 2 and S1). Consistent with the highest productivity of glycyrrhizin in Library 1, the highest expression of the 12 unigenes in Library 1 was also exhibited (Figures 2 and S1). In addition, the expression pattern of a different cytochrome P450 monooxygenase, *CYP93E3* (β‐amyrin 24‐oxidase), which is involved in the production of distinct oleanane‐type triterpenoids such as soyasapogenol B (aglycone of soyasaponin I and III; Figure S2; Seki *et al*., 2008), was also shown to be highly correlated with the 12 unigenes and glycyrrhizin biosynthetic genes (Figures 2 and S1). Therefore, it is possible that the 12 unigenes contain candidate UGTs involved in the biosynthesis of the soyasaponins as well as glycyrrhizin. As described in the Experimental procedures, we ultimately obtained eight full‐length coding sequences of UGTs from the 12 unigenes, and the eight candidate UGTs were then named by the UGT Nomenclature Committee as follows: *UGT72B31* (Unigene9884_All), *UGT73P12* (Unigene13257_All), *UGT91H11* (Unigene17749_All), *UGT73B27* (Unigene24516_All), *UGT73K3* (Unigene21536_All), *UGT73P13* (Unigene25821_All), *UGT87H4* (Unigene19049_All) and *UGT88E21* (Unigene22469_All/Unigene22470_All). Because the remaining unigenes (Unigene20598_All, Unigene27329_All and Unigene29474_All) contained only partial coding sequences, these unigenes were excluded from further analyses.

### Phylogenetic analysis of the candidate UGTs

**Figure 3:**
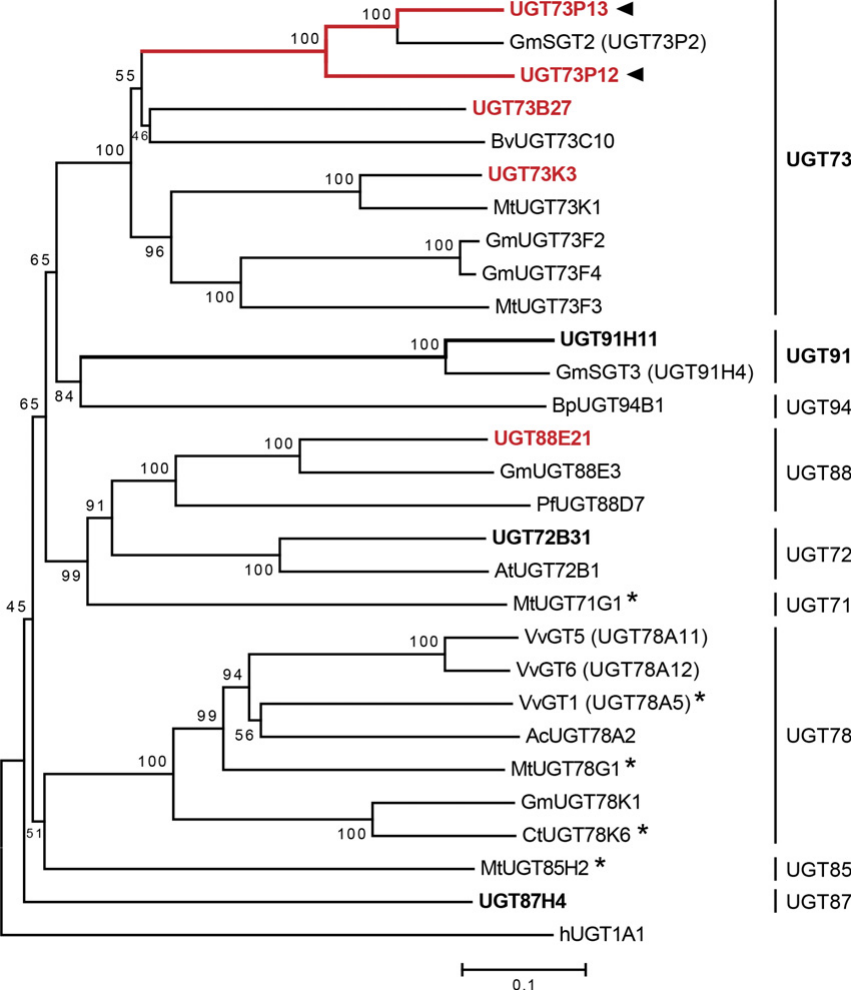
Phylogenetic relationships between candidate UGTs in *Glycyrrhiza uralensis* and their close relatives. The *bar* indicates 0.1 amino acid substitution per site. The *numbers* shown next to branches are the percentages of replicate trees in which the associated taxa clustered together in the bootstrap test (1000 replicates). The UGTs shown in bold font (black or red) are *G*. *uralensis* UGTs named in this study, and those shown in bold red font were further used to screen for functional proteins. *Arrowheads* indicate the UGTs characterized in this study. The names of close relatives that were previously characterized in other plants are tagged with the name of species abbreviated as follows: Gm, *Glycine max*; Bv, *Barbarea vulgaris*; Mt, *Medicago truncatula*; Bp, *Bellis perennis*; Pf, *Perilla frutescens*; At, *Arabidopsis thaliana*; Vv, *Vitis vinifera*; Ac, *Aralia cordata*; and Ct, *Clitoria ternatea*. The human (h) UGT1A1 was used as the outgroup. Superscript *stars* indicate the UDP‐glucose:flavonoid glucosyltransferases whose crystal structures are available.

We next elucidated phylogenetic relationships among the eight candidate UGT proteins and close relatives that had previously been characterized in other plants (Figure 3). Among the highly divergent members of plant UGTs, UGT73 family proteins, which share > 40% identity in their primary sequences, generally form the most common clade of the UGTs involved in the glycosylation of oleanane‐type triterpenoids (Augustin *et al*., 2011; Thimmappa *et al*., 2014; Seki *et al*., 2015). Therefore, we first selected the four UGT73 family proteins, UGT73B27, UGT73K3, UGT73P12 and UGT73P13, from the eight candidates. Of these, UGT73K3 was phylogenetically close to MtUGT73K1, which glucosylates multiple oleanane‐type triterpenoids, such as soyasapogenol B in *Medicago truncatula* (Achnine *et al*., 2005), implying the involvement of UGT73K3 in triterpenoid saponin biosynthesis. UGT73P13 showed remarkably high primary sequence identity (80%) and similarity (85%) to GmSGT2 (UGT73P2), which transfers a galactosyl moiety of UDP‐galactose to soyasapogenol B 3‐*O*‐monoglucuronide to produce soyasaponin III in *Glycine max* (Shibuya *et al*., 2010). Furthermore, the eight candidates included UGT91H11, which is a putative ortholog (79% identity and 84% similarity) of GmSGT3 (UGT91H4, *G*. *max* UDP‐rhamnose:soyasaponin III rhamnosyltransferase; Shibuya *et al*., 2010) and is likely involved in the production of soyasaponin I from soyasaponin III in *G. uralensis*. Given that the expression of *UGT73P13* is correlated with *CYP93E3* and *UGT91H11*, it is reasonable to assume that UGT73P13 is an ortholog of GmSGT2 and is involved in soyasaponin biosynthesis together with CYP93E3 and UGT91H11 in *G. uralensis*. The other UGT73P subfamily protein, UGT73P12, showed relatively low identity (64%) and similarity (71%) to GmSGT2 as compared with UGT73P13. Therefore, UGT73P12 is expected to be a paralog of UGT73P13 and a promising candidate for the glycyrrhizin biosynthetic protein in *G*. *uralensis*. Although the other candidates, including UGT88E21, did not show high similarity in the primary sequence with the previously characterized UGTs involved in the glycosylation of oleanane‐type triterpenoids, the expression pattern of *UGT88E21* showed the highest correlation with *CYP72A154* (Figures 2 and S1). We therefore selected UGT88E21. Finally, we selected the four candidates: UGT73B27, UGT73K3, UGT73P12 and UGT88E21. Additionally, UGT73P13 was also analyzed in this study to confirm the UDP‐galactose selectivity.

### Characterization of the candidate UGT proteins

**Figure 4:**
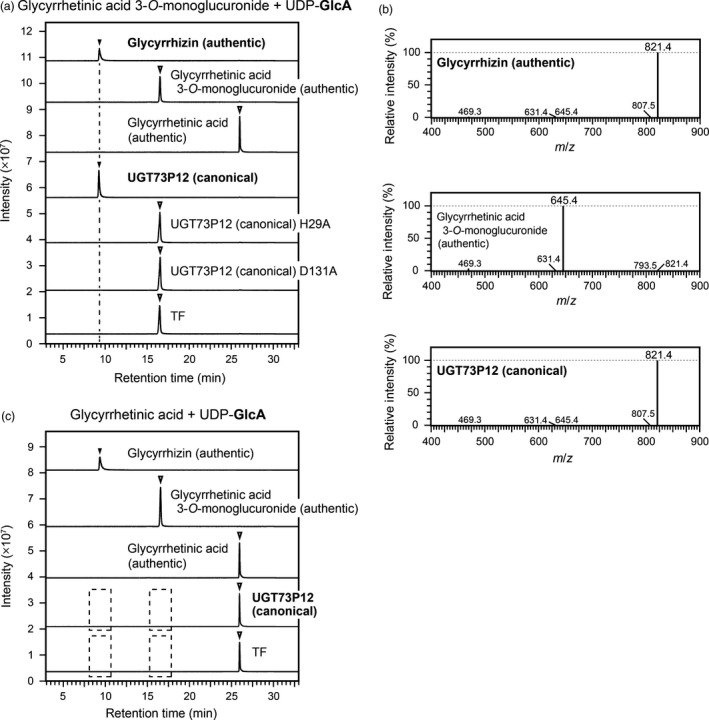
Catalytic activity of the canonical UGT73P12 protein. (a) Liquid chromatography‐mass spectrometry (LC‐MS) chromatogram of authentic compounds (glycyrrhetinic acid, glycyrrhetinic acid 3‐*O*‐monoglucuronide and glycyrrhizin) and *in vitro* reaction products. The enzyme reactions of the proteins isolated from *Escherichia coli*, including the canonical UGT73P12 and its two alanine‐scanning mutants (H29A and D131A), were individually performed in the presence of glycyrrhetinic acid 3‐*O*‐monoglucuronide as a sugar acceptor substrate and UDP‐glucuronic acid as a sugar donor, as described in the Experimental procedures. The reaction with trigger factor (TF) was used as a negative control. UDP‐glucuronic acid is abbreviated as UDP‐GlcA. (b) MS spectra of authentic compounds (glycyrrhetinic acid 3‐*O*‐monoglucuronide and glycyrrhizin) and the reaction product detected in (a) as the canonical UGT73P12 activity. (c) LC‐MS analysis of reaction mixtures. The enzyme reactions of the purified proteins, the canonical UGT73P12 and TF (negative control), were performed with the same reaction conditions as in (a), except for the presence of glycyrrhetinic acid as the sugar acceptor substrate. UDP‐glucuronic acid is abbreviated as UDP‐GlcA. Broken boxes indicate that no reaction product was detected. The LC‐MS data in (a–c) represent three independent reactions performed with different preparations of each purified protein.

**Figure 5:**
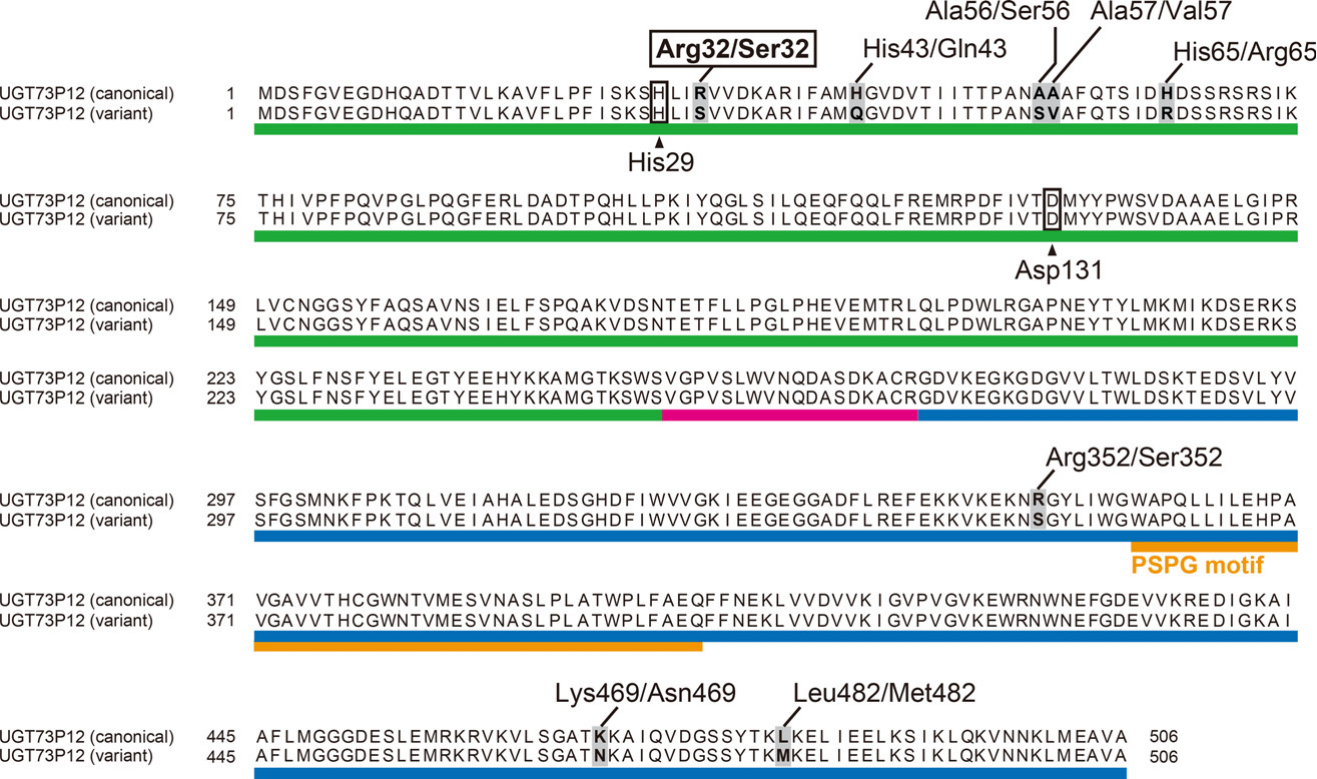
Comparison of the amino acid sequences between the canonical and variant UGT73P12 proteins. Light gray shading denotes the variant amino acid residues found between the canonical and variant UGT73P12 proteins. The catalytically essential residues, His29 and Asp131, are outlined in boxes. The N‐ and C‐domains and their linker region are underlined in green, blue and magenta, respectively. The plant secondary product glycosyltransferase (PSPG) motif sequence is underlined in orange.

To select functional proteins involved in glycyrrhizin biosynthesis from the four candidate UGTs, we targeted two types of glucuronosyltransferase activity, in which enzymes transfer a glucuronosyl moiety of UDP‐glucuronic acid into glycyrrhetinic acid and glycyrrhetinic acid 3‐*O*‐monoglucuronide to produce glycyrrhetinic acid 3‐*O*‐monoglucuronide and glycyrrhizin, respectively. We first prepared *Spodoptera frugiperda* 9 (Sf9) insect cells that were infected with recombinant baculoviruses expressing the candidate UGTs. This expression system assisted in the initial screening of the target enzymes because it enabled us to obtain the recombinant UGT proteins from the soluble fraction without the use of additional solubilization tags. Crude extracts of the Sf9 cells containing the candidate UGT proteins were initially used to screen for functional proteins, but only UGT73P12 protein showed one of the target enzymatic activities, i.e. the transfer of the glucuronosyl moiety from UDP‐glucuronic acid to glycyrrhetinic acid 3‐*O*‐monoglucuronide for glycyrrhizin production. Therefore, we expressed the recombinant UGT73P12 and its mutant proteins in *Escherichia coli* for further characterization. The soluble protein was isolated from the cells by cobalt‐immobilized metal affinity chromatography (Figure S3a), and its substrate preference *in vitro* was investigated. The reaction products were detected by high‐performance liquid chromatography‐mass spectrometer (LC‐MS). With the coexistence of glycyrrhetinic acid 3‐*O*‐monoglucuronide as a sugar acceptor substrate and UDP‐glucuronic acid as a sugar donor, the UGT73P12 protein yielded a single compound that corresponds to the authentic glycyrrhizin in terms of retention time (9.2 min) and exact mass of the deprotonated ion [M‐H]^–^ (*m*/*z *=* *821.4) (Figure 4a,b). However, two mutant proteins lacking His29 or Asp131 (Figures 5 and S4), both of which are essential for the catalytic function of UGT proteins (Shao *et al*., 2005), entirely lost their catalytic activity (Figure 4a). This confirmed that the catalytic function of UGT73P12 also depends on the typical residues, and the production of the compound is completely attributed to the catalytic function of the UGT73P12 protein. In addition, no reaction product was detected when incubated with glycyrrhetinic acid and UDP‐glucuronic acid (Figure 4c). These results therefore revealed that UGT73P12 can transfer the glucuronosyl moiety of UDP‐glucuronic acid to glycyrrhetinic acid 3‐*O*‐monoglucuronide to produce glycyrrhizin.

**Figure 6:**
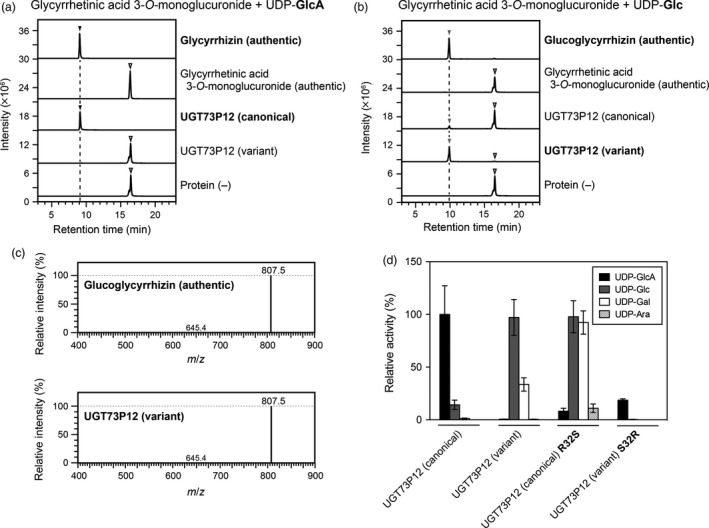
Catalytic activity of the variant UGT73P12 protein. (a,b) Liquid chromatography‐mass spectrometry (LC‐MS) chromatogram of authentic compounds (glycyrrhetinic acid 3‐*O*‐monoglucuronide, glycyrrhizin and glucoglycyrrhizin) and *in vitro* reaction products. The enzyme reactions of the purified canonical and variant UGT73P12 proteins were individually performed in the presence of glycyrrhetinic acid 3‐*O*‐monoglucuronide as the sugar acceptor substrate and (a) UDP‐glucuronic acid or (b) UDP‐glucose as the sugar donor (see Experimental procedures). A reaction without protein was used as a negative control. UDP‐glucuronic acid and UDP‐glucose are abbreviated as UDP‐GlcA and UDP‐Glc, respectively. (c) MS spectra of authentic glucoglycyrrhizin and the reaction product detected in (b) as the variant UGT73P12 activity. The LC‐MS data in (a–c) represent three separate reactions performed with the same preparation of each purified protein. (d) Comparison of UDP‐sugar selectivity among the canonical and variant UGT73P12 proteins and their residue‐swapped mutant proteins. The enzyme reactions of these purified proteins were individually performed under the same conditions as in (a) and (b) except for the inclusion of additional UDP‐sugars. The catalytic activity of the canonical UGT73P12 protein in the presence of UDP‐glucuronic acid was set as 100%. The following abbreviations are used: UDP‐GlcA, UDP‐glucuronic acid; UDP‐Glc, UDP‐glucose; UDP‐Gal, UDP‐galactose; and UDP‐Ara, UDP‐arabinose. The data are the mean ± SD of three independent reactions performed with the same preparation of each purified protein.

To check whether UGT73P12 has high specificity for UDP‐glucuronic acid as a sugar donor, we next performed enzymatic reactions of UGT73P12 protein (Figure S3b) with the sugar donors UDP‐glucose, UDP‐galactose and UDP‐arabinose (Figure 6d). Compared with the reactivity of the UGT73P12 protein in the presence of UDP‐glucuronic acid, the relative activities of the protein in the presence of UDP‐glucose and UDP‐galactose were only 14 and 1.3%, respectively. Moreover, no reaction product was detected in the presence of UDP‐arabinose. These results indicated that UGT73P12 preferentially selects UDP‐glucuronic acid as the sugar donor and is distinct from UGT73P13 specific for UDP‐galactose (Figure S5). Therefore, we defined this character as the canonical catalytic function of UGT73P12 protein.

To assess the catalytic efficiency (*k*_cat_/*K*_m_) of the canonical UGT73P12 in the presence of UDP‐glucuronic acid, the Michaelis–Menten kinetic parameters were also determined by quantifying the amount of reaction product in LC‐MS. The half‐maximal saturation concentration (Michaelis constant, *K*_m_) of the glycyrrhetinic acid 3‐*O*‐monoglucuronide, turnover number (*k*_cat_), and *k*_cat_/*K*_m_ values were 70.0 ± 21.8 μm, 1.92 ± 0.35 sec^−1^ and 28.3 ± 4.3 sec^−1^ mm ^−1^, respectively.

### Characterization of a natural variant of UGT73P12 derived from the glycyrrhizin‐deficient strain

The glycyrrhizin‐deficient strain 83‐555 accumulates glucoglycyrrhizin instead of glycyrrhizin (Figure 1; Hayashi *et al*., 2013). To evaluate the correlation between the production of glucoglycyrrhizin *in planta* and the sugar donor specificity of UGT73P12 protein derived from the 83‐555 strain, we first checked whether the 83‐555 strain harbors mutations in *UGT73P12*. Compared with the canonical UGT73P12, the 83‐555 strain‐derived UGT73P12 possessed 98% amino acid sequence identity and differed by only 8 of 506 amino acid residues (Figure 5). However, none of the variant residues was located within the PSPG motif.

To investigate the enzymatic function of the natural variant of UGT73P12, we next prepared the recombinant protein in *E. coli*. The variant UGT73P12 protein (Figure S3b) exhibited the highest activity in the presence of UDP‐glucose, resulting in the dominant production of glucoglycyrrhizin (retention time of 9.9 min and *m*/*z *=* *807.5; Figure 6). Compared with the canonical UGT73P12, the variant UGT73P12 displayed sevenfold and 27‐fold increases in the consumption of UDP‐glucose and UDP‐galactose, respectively, whereas the utilization of UDP‐glucuronic acid was drastically decreased to 0.6% (Figure 6d). Concomitantly, slight selectivity for UDP‐arabinose was also detected only in the variant UGT73P12. These results revealed that the difference in the eight residues between the canonical and variant UGT73P12 proteins is sufficient to change their UDP‐sugar selectivity. The kinetic parameters of the variant UGT73P12 in the presence of UDP‐glucose were then determined. The *K*_m_ of the glycyrrhetinic acid 3‐*O*‐monoglucuronide, *k*_cat_, and *k*_cat_/*K*_m_ values were 44.7 ± 21.5 μm, 0.25 ± 0.06 sec^−1^ and 6.45 ± 2.71 sec^−1^ mm ^−1^, respectively.

### Identification of an essential residue of the UGT73P12 protein to provide the high specificity for UDP‐glucuronic acid

**Figure 7:**
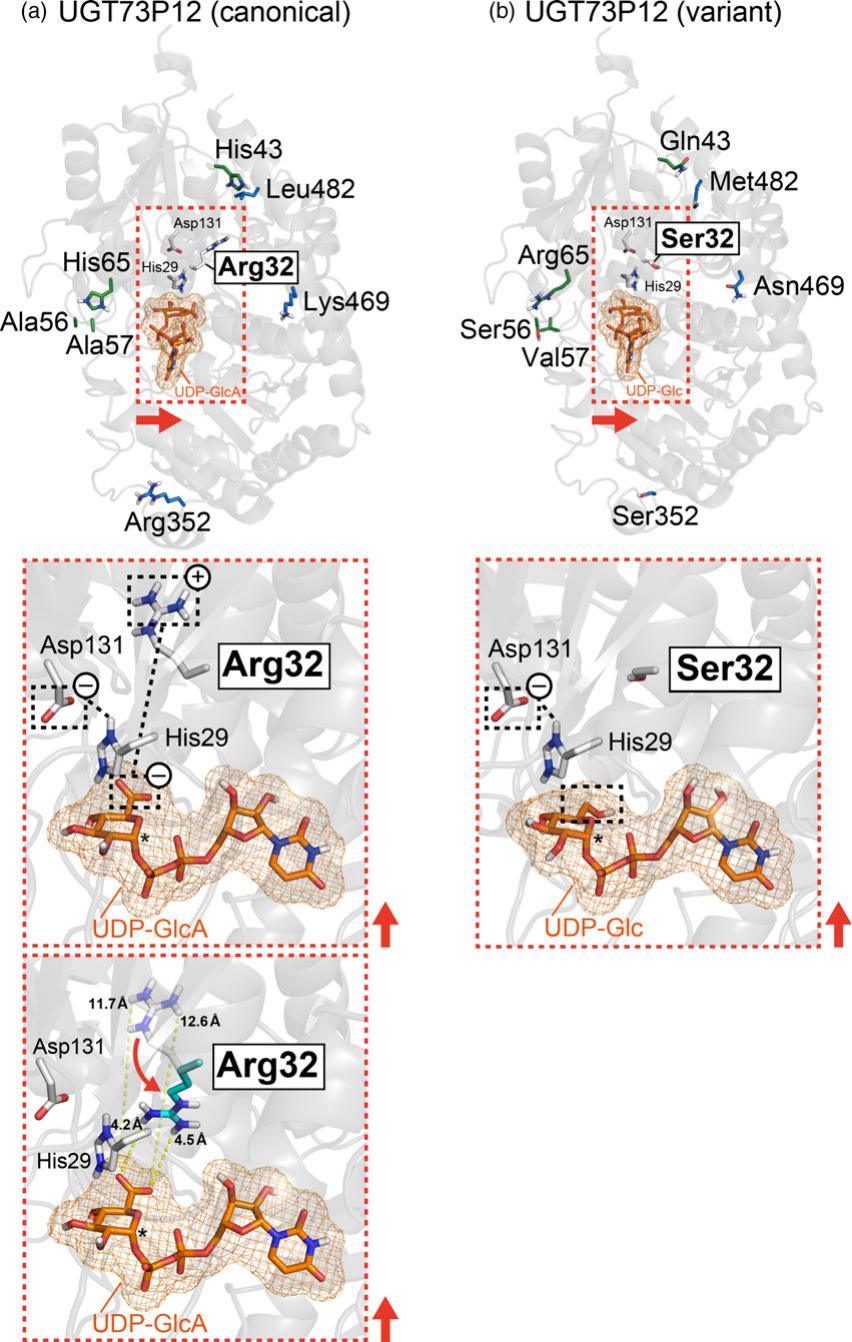
Virtual docking of UDP‐sugars onto homology models of UGT73P12 proteins. (a) Structural model of the canonical UGT73P12 protein docked with UDP‐glucuronic acid. (b) Structural model of variant UGT73P12 protein docked with UDP‐glucose. UDP‐glucuronic acid and UDP‐glucose are abbreviated as UDP‐GlcA and UDP‐Glc, respectively. The side‐chains of variant amino acid residues in the canonical and variant UGT73P12 proteins are shown in white (Arg32/Ser32; N‐domain residue), green (the other N‐domain residues) or blue (C‐domain residues) on each structural model (light gray background) (upper panels). The expected positions of sugar donor pockets in each protein are shown in the orange dotted box with the catalytically essential residues, His29 and Asp131 (N‐domain residues; white). The close‐up views (middle panels) indicate the predicted spatial arrangements of the key residues (His29, Asp131 and Arg32/Ser32) and UDP‐sugars (orange) within the sugar donor pockets in each protein. Possible electrostatic interactions among the key residues and UDP‐sugars are also shown with black dotted lines. The anomeric carbon of UDP‐sugars, which is related to the UGT reaction, is shown by *asterisks*. The direction of the red arrows in the overview maps was used to project the close‐up views. The possible conformation change of Arg32 in the canonical UGT73P12 protein and the minimal distance between the Arg32 and UDP‐glucuronic acid are displayed in the lower panel of (a).

Homology modeling of three‐dimensional protein structures provides further insights into the structural basis of UGT proteins (Osmani *et al*., 2009; Sharma *et al*., 2014; Itkin *et al*., 2016). Therefore, we constructed homology models of the canonical and variant UGT73P12 proteins using the automated SWISS‐MODEL pipeline (Biasini *et al*., 2014) and estimated the spatial arrangements of the eight variant residues within the three‐dimensional structure of the UGT73P12 proteins. The crystal structure of MtUGT71G1 protein (PDB ID: 2ACW chain A; Shao *et al*., 2005) was selected as a reliable template structure. The constructed models of the canonical and variant UGT73P12 proteins were identical, except for the eight variant residues (Figure 7). The catalytically essential residues, His29 and Asp131, were positioned inside the proteins. Interestingly, only one variant residue at position 32, which corresponds to Arg32 and Ser32 in the canonical and variant UGT73P12 proteins, respectively, was located inside the protein with the residues forming a catalytic site, whereas the other seven variant residues were scattered on the protein surface. In addition, Arg32/Ser32 was adjacent to the His29‐Asp131 pair in the models.

To estimate further the spatial position of the sugar donor pocket in the UGT73P12 proteins, we performed virtual docking of UDP‐glucuronic acid and UDP‐glucose onto the homology models of the canonical and variant UGT73P12 proteins, respectively, with AutoDock Vina (Trott and Olson, 2010). The reliability of the virtual molecular docking was preliminarily evaluated by comparing the simulated position of UDP‐glucose in the MtUGT71G1 protein with the crystal data of MtUGT71G1 protein bound to UDP‐glucose (PDB ID: 2ACW chain A; Shao *et al*., 2005). The simulation precisely predicted not only the position but also the orientation of UDP‐glucose in the MtUGT71G1 protein (Figure S6). We then investigated UGT73P12. The docking models of the canonical and variant UGT73P12 proteins were identical in the position and orientation of UDP‐sugars (Figures 7 and S7). The sugar moiety of the UDP‐sugars was positioned near the His29‐Asp131 pair and Arg32/Ser32. To consider the possible conformation change of Arg32, which has a side‐chain that possesses high conformational flexibility, we surveyed virtual rotamers of the Arg32 and found a minimal distance of 4.2 Å between the flexible side‐chain of Arg32 and a carboxy group of UDP‐glucuronic acid in the model (Figure 7).

Collectively, the computational modeling clearly suggested that only Arg32/Ser32 can directly access the sugar moiety of UDP‐sugars among the eight variant residues, and Arg32 and Ser32 may primarily define the UDP‐sugar selectivity of the canonical and variant UGT73P12 proteins, respectively. To address this hypothesis, we swapped Arg32 and Ser32 between the canonical and variant UGT73P12 proteins by site‐directed mutagenesis. The purified canonical UGT73P12 protein harboring the R32S mutation (Figure S3b) largely lost the specificity for UDP‐glucuronic acid and gained high selectivity for UDP‐glucose and UDP‐galactose (Figure 6d). This sugar donor preference is consistent with the variant UGT73P12 (Figure 6d). Compared with the original canonical UGT73P12 protein, the protein harboring the R32S mutation displayed sevenfold and 73‐fold increases in the consumption of UDP‐glucose and UDP‐galactose, respectively, whereas the utilization of UDP‐glucuronic acid was markedly decreased to 8.2%. Weak selectivity for UDP‐arabinose was also observed in the mutant protein. We then investigated the variant UGT73P12 protein harboring the S32R mutation (Figure S3b), and found that this mutation is sufficient to recover the high specificity for UDP‐glucuronic acid and to abolish the selectivity for UDP‐glucose and UDP‐galactose (Figure 6d). These results therefore revealed that Arg32 is the key residue conferring the precise selectivity for UDP‐glucuronic acid on the UGT73P12 protein.

**Figure 8:**
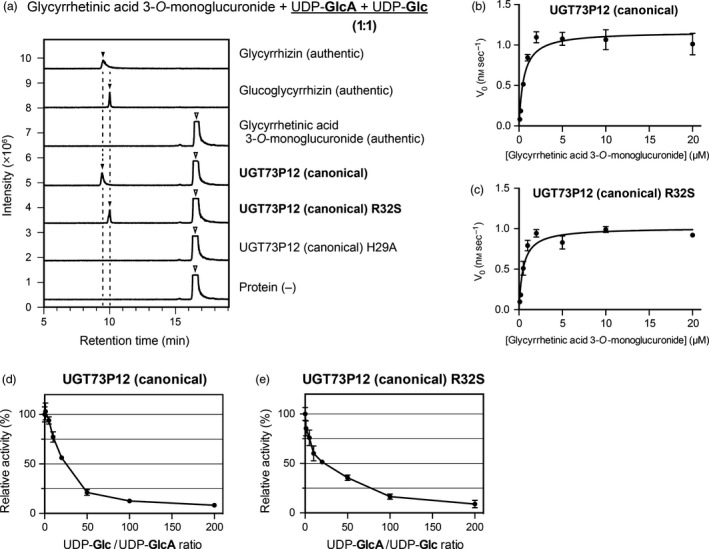
UDP‐sugar competition experiments on the UGT73P12 protein. (a) Liquid chromatography‐mass spectrometry (LC‐MS) chromatogram of authentic compounds (glycyrrhetinic acid 3‐*O*‐monoglucuronide, glycyrrhizin and glucoglycyrrhizin) and *in vitro* reaction products. The enzyme reactions of purified proteins, including the canonical UGT73P12 and its two mutants (H29A and R32S), were individually performed in the presence of glycyrrhetinic acid 3‐*O*‐monoglucuronide as the sugar acceptor substrate and two competitive sugar donors, UDP‐glucuronic acid and UDP‐glucose (1:1), as described in the Experimental procedures. A reaction without protein was included as a negative control. UDP‐glucuronic acid and UDP‐glucose are abbreviated as UDP‐GlcA and UDP‐Glc, respectively. The LC‐MS data represent three independent reactions performed with the same preparation of each purified protein. (b,c) Michaelis–Menten curves of (b) the canonical UGT73P12 and (c) its R32S mutant protein. The enzyme reactions of these proteins were individually performed in the presence of both UDP‐glucuronic acid and UDP‐glucose (1:1), as described in the Experimental procedures. The data are the means ± SD of three separate reactions performed with the same preparation of each purified protein. (d,e) Inhibitory effect of (d) UDP‐glucose and (e) UDP‐glucuronic acid on the catalytic activity of (d) the canonical UGT73P12 and (e) its R32S mutant protein. The catalytic activity of the canonical UGT73P12 and its R32S mutant protein in the absence of UDP‐glucose and UDP‐glucuronic acid, respectively, was set as 100%. The data are the means ± SD of three separate reactions performed with the same preparation of each purified protein.

To further evaluate the extent to which the Arg32 confers the high specificity for UDP‐glucuronic acid in the UGT73P12 protein, we performed enzymatic reactions of the canonical UGT73P12 and its R32S mutant protein in the presence of two competitive sugar donors, UDP‐glucuronic acid and UDP‐glucose, at the same concentrations. Even in the presence of UDP‐glucuronic acid and UDP‐glucose, the canonical UGT73P12 yielded a single product, glycyrrhizin, while the protein harboring the R32S mutation produced glucoglycyrrhizin instead of glycyrrhizin (Figure 8a). These sugar donor preferences were compatible with those observed in the presence of a single sugar donor (Figure 6d). These results confirmed that Arg32 conferred the high degree of specificity for UDP‐glucuronic acid on the UGT73P12 protein. To assess the impact of Arg32 on the catalytic efficiency (*k*_cat_^app^/*K*_m_^app^) of the UGT73P12 protein, we determined the apparent kinetic parameters (*K*_m_^app^ of glycyrrhetinic acid 3‐*O*‐monoglucuronide, *k*_cat_^app^, and *k*_cat_^app^/*K*_m_^app^) of the canonical UGT73P12 and its R32S mutant protein with the same concentration of UDP‐glucuronic acid and UDP‐glucose (Table 1) from the Michaelis–Menten curves (Figure 8b,c). In accordance with the results of Figure 8(a), the canonical UGT73P12 and its R32S mutant protein each produced only a single product, glycyrrhizin and glucoglycyrrhizin, respectively, regardless of the concentration of glycyrrhetinic acid 3‐*O*‐monoglucuronide. Therefore, the initial velocities (*V*_0_) of glycosyltransferase activity catalyzed by the canonical UGT73P12 and its R32S mutant protein were essentially equal to the productivity of glycyrrhizin and glucoglycyrrhizin, respectively (Figure 8b,c). Similar saturation curves were obtained from the Michaelis–Menten curves of the canonical UGT73P12 protein and its R32S mutant protein (Figure 8b,c); therefore, their *k*_cat_^app^/*K*_m_^app^ values were also equivalent (Table 1). These results indicate that Arg32 has no apparent effect on the catalytic efficiency of the UGT73P12 protein and contributes only to the determination of the sugar donor preference in the UGT73P12 protein. Notably, the *K*_m_^app^ and *k*_cat_^app^ values, which were calculated in the presence of one original sugar donor substrate and another potential competitive sugar donor, were markedly decreased when compared with the *K*_m_ and *k*_cat_ values only in the presence of the original sugar donor substrate. The difference between the *k*_cat_^app^/*K*_m_^app^ and *k*_cat_/*K*_m_ ratios was not considered here because of the large difference between the *K*_m_^app^ and *K*_m_ values and the resultant usage limit of the *k*_cat_^app^/*K*_m_^app^ ratio, as previously described (Koshland, 2002; Eisenthal *et al*., 2007; Carrillo *et al*., 2010). The reduction of the *K*_m_^app^ and *k*_cat_^app^ values indicates mixed or uncompetitive inhibition of UGT73P12 protein activity by the potential competitive sugar donor. In other words, the potential competitive sugar donor suggests that it does not bind to the binding site of glycyrrhetinic acid 3‐*O*‐monoglucuronide but instead binds to other enzyme sites. Given that UGT73P12 protein catalyzes glycosyltransferase activity with a bi‐substrate (sugar acceptor and donor substrates) sequential mechanism (i.e. single‐displacement mechanism), it is reasonable to assume that the potential competitive sugar donor, the analog of the original sugar donor, binds to the sugar donor binding site but not to the sugar acceptor binding site. Accordingly, we further tested whether the potential competitive sugar donor exhibited an inhibitory effect on the catalytic activity of the UGT73P12 protein by competing with the original sugar donor substrate. Increasing the concentration of the potential competitive sugar donor UDP‐glucose against a constant concentration of the original sugar donor substrate UDP‐glucuronic acid inhibited the catalytic activity of the canonical UGT73P12 protein (Figure 8d). Concomitantly, a trace amount of byproduct, glucoglycyrrhizin, was detected only when supplemented with UDP‐glucose concentrations > 100 times those of UDP‐glucuronic acid (Figure S8a). Similarly, the catalytic activity of the protein harboring the R32S mutation was inhibited by increasing the concentration of the potential competitive sugar donor UDP‐glucuronic acid compared with a constant concentration of the original sugar donor substrate UDP‐glucose (Figure 8e). In contrast to the canonical UGT73P12 protein, the R32S mutant did not produce glycyrrhizin as a byproduct, even when supplemented with UDP‐glucuronic acid concentrations > 100 times those of UDP‐glucose (Figure S8b). Taken together, our results reveal that Arg32 has a pivotal role in the UGT73P12 protein in controlling UDP‐glucuronic acid use.

**Table 1:** Kinetic parameters of UGT73P12 proteins in the presence of both UDP‐glucuronic acid and UDP‐glucose (1:1)

Enzyme	Glycyrrhetinic acid 3‐*O*‐monoglucuronide			Reaction product
	*K*_m_^app^ (μm)	*k*_cat_^app^ (sec^−1^)	*k*_cat_^app^/*K*_m_^app^ (sec^−1^ mm ^−1^)	
UGT73P12 (canonical)	0.55 ± 0.08	0.058 ± 0.006	107 ± 6	Glycyrrhizin
UGT73P12 (canonical) R32S	0.49 ± 0.06	0.051 ± 0.002	105 ± 16	Glucoglycyrrhizin

Data are the mean ± SD of three repeated experiments as described in the Experimental procedures.

## Discussion

In this study, we characterized the canonical and variant UGT73P12 proteins in the glycyrrhizin‐producing and ‐deficient (83‐555) strains, respectively. The canonical UGT73P12 protein transferred the glucuronosyl moiety of UDP‐glucuronic acid to glycyrrhetinic acid 3‐*O*‐monoglucuronide to produce glycyrrhizin. The specificity for UDP‐glucuronic acid is remarkably high, thereby ensuring the production of glycyrrhizin. By contrast, the variant UGT73P12 protein lacked the specificity for the UDP‐glucuronic acid and produced glucoglycyrrhizin. The kinetic parameters indicated that the effective catalytic activities of the canonical and variant UGT73P12 proteins were similar to those of previously characterized UGTs in other plants (Sayama *et al*., 2012). More importantly, our biochemical results were consistent with the production of glycyrrhizin and glucoglycyrrhizin in the glycyrrhizin‐producing and ‐deficient (83‐555) strains, respectively. Based on these results, we suggest the contribution of UGT73P12 to glycyrrhizin biosynthesis *in planta*. Our transcriptome data also support this view because the data showed that *UGT73P12* is expressed exclusively in the roots together with the other glycyrrhizin biosynthetic genes, *bAS*,* CYP88D6* and *CYP72A154*, and this expression pattern is compatible with the pattern of accumulation of glycyrrhizin.

Our results further confirmed that UGT73P12 has no catalytic activity in the presence of glycyrrhetinic acid as a sugar acceptor substrate. Therefore, it is reasonable to propose the existence of a separate enzyme responsible for the first glucuronosylation of glycyrrhetinic acid to produce glycyrrhetinic acid 3‐*O*‐monoglucuronide (Figure 1). Recently, Xu *et al*. reported that GuUGAT, which corresponds to UGT73B27 in our study but differs from UGT73B27 by one amino acid residue (Leu329 in GuUGAT versus Phe329 in UGT73B27), has catalytic activity to produce glycyrrhizin directly from glycyrrhetinic acid through a continuous two‐step glucuronosylation reaction (Xu *et al*., 2016). The target enzymatic activity was, however, not detected in UGT73B27 in our assay (Figure S9). Furthermore, we also prepared a protein identical to GuUGAT through F329L mutation of UGT73B27, and performed the enzyme assay. However, no significant catalytic activity of the GuUGAT protein was detected in our assay (Figure S9). As described above, our results for UGT73P12 agree perfectly with the chemical phenotype of the 83‐555 strain, and strongly suggest the contribution of the UGT73P12 to glycyrrhizin biosynthesis *in planta*. The sugar donor specificity of GuUGAT in the 83‐555 strain has not been investigated. Therefore, in addition to the catalytic activity of GuUGAT, the contribution of GuUGAT to glycyrrhizin biosynthesis in *G. uralensis* remains controversial. To address these questions further, it is necessary to establish transgenic systems to perform knockdown and/or knockout of the target genes in licorice plants in the future.

In this study, the 83‐555 strain further served to identify Arg32 as the essential residue of the UGT73P12 protein that provides the high specificity for UDP‐glucuronic acid. In fact, our residue‐swapping experiments successfully demonstrated that the lack of Arg32 leads to loss of the specificity for the UDP‐glucuronic acid in the canonical UGT73P12, and the introduction of Arg32 confers high specificity for UDP‐glucuronic acid on the variant UGT73P12. These results indicate the plasticity of UGT73P12 in UDP‐sugar specificity. In addition, the results strongly suggest a model for the molecular mechanism in which a positively charged side‐chain (guanidino group) of Arg32 is oriented toward a negatively charged carboxy group of UDP‐glucuronic acid due to their electrostatic interaction, and the resultant salt bridge, including hydrogen bonding, provides high affinity for UDP‐glucuronic acid in UGT73P12 (Figure 7). In general, substrate‐induced conformational changes of glycosyltransferases further increase the accessibility of amino acid side‐chains to substrates and ensure their strong interaction (Qasba *et al*., 2005). These protein dynamics were not investigated in this study, but the computer‐simulated distance of 4.2 Å between the side‐chain of Arg32 and the carboxy group of UDP‐glucuronic acid (with a distance of 4.7 Å between their N and O atoms) in the static homology model implies the existence of non‐covalent interactions (Kumar and Nussinov, 2002; Cohen *et al*., 2009).

Similarly, previous works have identified key residues to define UDP‐sugar selectivity in flavonoid UGTs. As one of the relevant examples, three UDP‐glucuronic acid:flavonoid glucuronosyltransferases derived from *Bellis perennis* (BpUGT94B1), *Vitis vinifera* (VvGT5; UGT78A11) and the Lamiales (e.g. PfUGT88D7) are known to have independently acquired a functionally equivalent arginine residue in three different positions during convergent evolution, and the lack of these arginine residues causes a shift in sugar donor specificity from UDP‐glucuronic acid to UDP‐glucose (Osmani *et al*., 2008; Noguchi *et al*., 2009; Ono *et al*., 2010). This example is particularly interesting because the acquisition of an arginine residue and resultant formation of a salt bridge between the arginine residue and UDP‐glucuronic acid seem to be a common event leading to a gain in specificity for UDP‐glucuronic acid during convergent evolution not only in flavonoid UGTs but also in triterpenoid UGTs. Intriguingly, we found that Arg32 of UGT73P12 and Arg25 of BpUGT94B1 are in the same position in the alignment of their primary sequences (Figure S4). This coincidence supports the view that the flavonoid UGTs and triterpenoid UGTs share high similarity in protein structure. In fact, homology models of UGT73P12 and BpUGT94B1 had nearly identical spatial arrangements of the His‐Asp pair, arginine residue and UDP‐glucuronic acid within the catalytic site (Figure S10). In addition, the other arginine residues found in VvGT5 and PfUGT88D7 (Arg140 and Arg350, respectively) were predicted to be close to the His‐Asp pair and the carboxy group of UDP‐glucuronic acid in homology models, even though the positions of these arginine residues differed from those of UGT73P12 and BpUGT94B1 in the primary sequence (Noguchi *et al*., 2009; Ono *et al*., 2010). The appearance of an arginine residue in such limited positions further implies that these UGTs are limited in the available positions for the arginine residue within the catalytic site and their arginine residues are functionally equivalent.

Besides the key role of the arginine residue in the utilization of UDP‐glucuronic acid, positive effects of the arginine residue on the catalytic efficiency (*k*_cat_/*K*_m_) of BpUGT94B1, VvGT5 and PfUGT88D7 have been observed (Osmani *et al*., 2008; Noguchi *et al*., 2009; Ono *et al*., 2010), especially in PfUGT88D7. Conversely, our results indicated no apparent effect of Arg32 on the catalytic efficiency of UGT73P12. These observations imply the increased dependence of the catalytic activity of BpUGT94B1, VvGT5 and PfUGT88D7 on their arginine residues. In other words, these flavonoid UGTs appear to have lost the residues required to bind with high affinity to UDP‐sugars, except UDP‐glucuronic acid, and/or have acquired additional unique residues that increase the specificity for UDP‐glucuronic acid through relatively long‐term evolution after the acquisition of the arginine residue. Interestingly, a serine residue (Ser127) found in PfUGT88D7 was shown to further increase the specificity for UDP‐glucuronic acid, perhaps via hydrogen bonding (Noguchi *et al*., 2009). An equivalent serine residue was found in BpUGT94B1 (Ser143), but not in UGT73P12 (Figure S4). Sayama *et al*. (2012) also reported the requirement of the equivalent serine residue (Ser138) in GmUGT73F4 for its strict UDP‐xylose specificity, supporting the importance of these serine residues in UDP‐sugar specificity. Meanwhile, the catalytic activity of UGT73P12 was independent of its Arg32. As such, UGT73P12 has high plasticity in UDP‐sugar specificity, as opposed to flavonoid UGTs. This suggests the relatively recent acquisition of Arg32 in UGT73P12. Consequently, the catalytic mechanism of UGT73P12 is likely to be similar to those of flavonoid UGTs.

The catalytic mechanism of flavonoid UGTs is explained by a single‐displacement S_N_2 mechanism, in which the histidine residue of the His‐Asp pair deprotonates the hydroxy group of sugar acceptors, and the resulting nucleophilic oxyanion of sugar acceptors attacks an anomeric carbon of UDP‐sugars to obtain their sugar moiety (Shao *et al*., 2005; Offen *et al*., 2006). In this mechanism, a hydrogen bond between the His‐Asp pair is required to activate the catalytic histidine residue as a general base. In addition, we need to pay attention to the behavior of the arginine residue against the His‐Asp pair in UGTs harboring the arginine residue (Ono *et al*., 2010). In the presence of UDP‐glucuronic acid as a sugar donor, the hydrogen bond between the His‐Asp pair is retained because the positively charged side‐chain of the arginine residue can preferentially interact with the negatively charged carboxy group of UDP‐glucuronic acid (Figures 7 and S10). However, in the presence of an electrically neutral sugar moiety of UDP‐sugars, including UDP‐glucose, UDP‐galactose and UDP‐arabinose, the positively charged side‐chain of the arginine residue interacts alternatively with a negatively charged side‐chain of the aspartate residue of the His‐Asp pair and interrupts the hydrogen bond. This model explains why UGT73P12 can repress the selectivity against the electrically neutral sugar moiety of UDP‐sugars. Furthermore, the UDP‐sugar competition experiments indicated the existence of an inhibitory effect of UDP‐glucose on the catalytic activity of UGT73P12 and the limited utilization of UDP‐glucose as a sugar donor in UGT73P12 (Figures 8d and S8a). This model further suggests that UGTs lacking the arginine residue not only lose their high affinity for UDP‐glucuronic acid but also permit the negatively charged carboxy group of UDP‐glucuronic acid to interact with a positively charged side‐chain of the catalytic histidine residue, leading to the inactivation of UGTs. In support of this view, our UDP‐sugar competition experiments demonstrated the inhibitory effects of UDP‐glucuronic acid on the catalytic activity of the mutant UGT73P12 lacking Arg32, albeit at high concentrations of UDP‐glucuronic acid, due to the drastic reduction in the binding affinity for UDP‐glucuronic acid (Figure 8e). Therefore, the arginine residue seems to be essential to stabilize the UDP‐glucuronic acid in the catalytic site of UGTs. In fact, the other UGT73P subfamily protein, UGT73P13, possesses a proline residue (Pro25) instead of the arginine residue (Figure S4), and completely lacks specificity for UDP‐glucuronic acid (Figure S5). This observation is a good example of the plasticity of the UGT73P subfamily proteins in UDP‐sugar selectivity and highlights the functional specialization of UGT73P12 in UDP‐glucuronic acid for glycyrrhizin biosynthesis during divergent evolution (Figure S11).

The sugar moieties of triterpenoid saponins affect their taste, solubility, stability and pharmacological efficacy. Hence, chemical modification of the sugar moiety of glycyrrhizin and the enzymatic transglycosylation of licorice extracts have been investigated to improve their taste (Liu *et al*., 2000; Mizutani *et al*., 2014). Protein engineering is now a promising approach to switch the sugar donor specificity of UGTs and to design novel sweet triterpenoid saponins as newer non‐caloric sweeteners. In this study, we found intriguing evidence of the structural similarity of flavonoid and triterpenoid UGTs. This finding suggests the validity of the three‐dimensional homology‐based approach using the crystal data of flavonoid UGT proteins as template proteins when we first attempt to consider the molecular mechanisms of triterpenoid UGTs for protein engineering. Among the plant UGTs, however, only five crystal structures of UDP‐glucose:flavonoid glucosyltransferases are available: *V*.* vinifera* VvGT1 (UGT78A5), *M*.* truncatula* UGT71G1, UGT78G1 and UGT85H2, and *Clitoria ternatea* UGT78K6 (Shao *et al*., 2005; Offen *et al*., 2006; Li *et al*., 2007; Modolo *et al*., 2009; Hiromoto *et al*., 2013). Therefore, the crystal analyses of triterpenoid UGT proteins that are bound to triterpenoids and UDP‐sugars (especially, UDP‐glucuronic acid) are still desired to improve their structural prediction using homology modeling and to understand more precisely the determinants of UDP‐sugar selectivity. These future works might open the possibility for the rational design of UGT functions and sweet triterpenoid saponins. Again, this structure‐guided engineering of UGT proteins should facilitate the future reconstitution of tailor‐made biosynthetic pathways in heterologous hosts, such as microbes and domesticated plants, for the rapid, inexpensive production of high‐value natural and new‐to‐nature saponins (Moses *et al*., 2014; Arendt *et al*., 2016; Reed *et al*., 2017).

## Experimental procedures

### Gene annotation and expression analysis

A dataset of non‐redundant unigene sequences and their expression profiles was previously obtained by RNA‐Seq of *G*. *uralensis* cDNA libraries (Ramilowski *et al*., 2013). Annotation of putative UGTs and unigene expression analysis were performed as previously established (Ramilowski *et al*., 2013; Mochida *et al*., 2017), with minor modifications (see Method S1 for details).

### Plasmid construction

Full‐length coding sequences of the eight candidate UGTs, *UGT72B31*,* UGT73B27*,* UGT73K3*,* UGT73P12*,* UGT73P13*,* UGT87H4*,* UGT88E21* and *UGT91H11*, were retrieved from the 12 unigene sequences, as described in Method S2. The corresponding coding regions were then amplified by polymerase chain reaction (PCR) from cDNA of *G. uralensis* with gene‐specific primers, 1–18, as described in Method S3. Primers for PCR are listed in Table S1. These PCR products were then cloned directly into a pENTR/D‐TOPO entry vector (Invitrogen, Carlsbad, CA, USA), as previously described (Muangphrom *et al*., 2014). Sequence determination of these genes within the entry clones was then performed. Site‐directed mutagenesis of *UGT73P12* was performed in the entry clone by inverse PCR with the gene‐specific primers, 19–26. Expression vectors were produced by an LR clonase reaction between the entry clones and a modified pColdTF vector (Novagen, Madison, WI, USA) harboring a gateway cassette (Muangphrom *et al*., 2014).

### Phylogenetic analysis

Reference sequences were obtained from GenBank or Swiss‐Prot (http://www.uniprot.org) databases. The protein sequences were aligned using the GenomeNet ClustalW 1.83 server (http://www.genome.jp/tools-bin/clustalw), and the result was colored using the BoxShade 3.21 server (https://embnet.vital-it.ch/software/BOX_form.html). A neighbor‐joining phylogenetic tree was then constructed using the p‐distance algorithm in MEGA 7.0 (Kumar *et al*., 2016) with 1000 bootstrap replicates.

### Expression and purification of UGT proteins

All recombinant UGT proteins were expressed in *E*.* coli* Rosetta 2(DE3)pLysS (Novagen) and purified with the use of TALON metal affinity resin (Clontech, Mountain View, CA, USA), as described in Method S4. The concentration of these purified proteins was determined using the protein assay kit (Bio‐Rad, Richmond, CA, USA) with bovine serum albumin as a standard.

### Glycosyltransferase assay

Authentic samples, including (18β‐)glycyrrhetinic acid (Extrasynthese, Genay, France), glycyrrhetinic acid 3‐*O*‐monoglucuronide (Nacalai Tesque, Kyoto, Japan), glycyrrhizin (Wako Pure Chemical Industries, Osaka, Japan), glucoglycyrrhizin (Hayashi *et al*., 2013), soyasapogenol B (Tokiwa Phytochemical, Chiba, Japan), soyasapogenol B 3‐*O*‐monoglucuronide (Yano *et al*., 2018) and soyasaponin III (ChromaDex, Santa Ana, CA, USA), were used for the enzyme assay and/or LC‐MS analysis. For the initial characterization of the canonical UGT73P12 protein (WT, H29A and D131A) and the UGT73P13 protein, the reaction was initiated with the addition of 5 μl of purified protein (final concentration: 200 nm) into 95 μl of a solution containing 50 mm Tris‐HCl (pH 7.0), 100 μm MgCl_2_, 14 mm 2‐mercaptoethanol, 10 μm sugar acceptors and 50 μm UDP‐sugars. The mixture was incubated at 30°C for 1 h. To obtain detailed characterization of the canonical UGT73P12 protein (WT and R32S) and variant UGT73P12 protein (WT and S32R) with respect to their sugar donor selectivity, the reaction was performed in a total volume of 200 μl of reaction mixture containing 50 mm Tris‐HCl (pH 7.0), 10 mm MgCl_2_, 14 mm 2‐mercaptoethanol, 600 μm glycyrrhetinic acid 3‐*O*‐monoglucuronide, 5 mm UDP‐sugars and 100 nm purified proteins at 30°C for 1 h. For the kinetic assay of the canonical and variant UGT73P12 proteins in the presence of a single sugar donor, several reaction conditions were tested: a total volume of 100 μl of reaction mixture containing 50 mm Tris‐HCl (pH 7.0), 10 mm MgCl_2_, 14 mm 2‐mercaptoethanol, 10–500 μm glycyrrhetinic acid 3‐*O*‐monoglucuronide, 2 mm UDP‐sugars and 177 nm purified proteins at 30°C for 0–30 min. For the UDP‐sugar competition experiments of the canonical UGT73P12 protein (WT, R32S and H29A), the reaction was initiated with the addition of 5 μl of purified proteins (final concentration: 20 nm) into 95 μl of a solution containing 50 mm Tris‐HCl (pH 7.0), 100 μm MgCl_2_, 14 mm 2‐mercaptoethanol, 10 μm glycyrrhetinic acid 3‐*O*‐monoglucuronide, 1 mm UDP‐glucuronic acid and 1 mm UDP‐glucose. The mixture was then incubated at 30°C for 10 min. For the kinetic assay of the canonical UGT73P12 protein (WT and R32S) in the presence of two sugar donors, several reaction conditions were tested: a total volume of 100 μl of reaction mixture containing 50 mm Tris‐HCl (pH 7.0), 100 μm MgCl_2_, 14 mm 2‐mercaptoethanol, 0.1–500 μm glycyrrhetinic acid 3‐*O*‐monoglucuronide, 1 mm UDP‐glucuronic acid, 1 mm UDP‐glucose and 20 nm purified proteins at 30°C for 0–10 min. For the assessment of the inhibitory effect of potential competitive UDP‐sugars on the catalytic activity of the canonical UGT73P12 protein (WT and R32S), the reaction was initiated with the addition of 5 μl of purified proteins (final concentration: 20 nm) into 95 μl of a solution containing 50 mm Tris‐HCl (pH 7.0), 100 μm MgCl_2_, 14 mm 2‐mercaptoethanol, 10 μm glycyrrhetinic acid 3‐*O*‐monoglucuronide, and several concentrations of UDP‐sugars as follows: 10 μm UDP‐glucuronic acid and 0–2000 μm UDP‐glucose for the canonical UGT73P12 protein (WT), and 10 μm UDP‐glucose and 0–2000 μm UDP‐glucuronic acid for the canonical UGT73P12 protein (R32S). The mixture was then incubated at 30°C for 10 min. These reactions were terminated by freezing in liquid nitrogen or adding 1‐butanol, and the solution, supplemented with 1‐butanol, was centrifuged at 100 ***g*** for 5 min at 4°C. The resulting supernatant was evaporated with a centrifugal evaporator. Then the dried material was dissolved in methanol and the solution was passed through a GL chromate disk 4A filter (pore size: 0.2 μm; GL Sciences, Tokyo, Japan) to remove debris. The resulting samples were used for the LC‐MS analysis.

### Liquid chromatography‐mass spectrometry analysis

The reaction products were detected with the ACQUITY UPLC/MS system (Waters, Milford, MA, USA) in electrospray ionization negative ion mode with selected ion monitoring, as described in Method S5. The amount of reaction products was determined as the peak area using MassLynx software (Waters). The kinetic parameters were calculated by fitting a Michaelis–Menten curve to the raw kinetic data using GraphPad Prism 6.0h software (GraphPad Software, San Diego, CA, USA). The raw data were calibrated beforehand with standard curves of authentic glycyrrhizin and glucoglycyrrhizin.

### Virtual docking of UDP‐sugars onto homology models of UGT proteins

Three‐dimensional structures of ligands, UDP‐glucuronic acid and UDP‐glucose, were constructed by Avogadro 1.2.0 software (Hanwell *et al*., 2012). The geometries of the structures were refined to minimize their free energy state using Avogadro. The three‐dimensional structures of the UGT73P12 and BpUGT94B1 proteins were predicted by the SWISS‐MODEL pipeline (Biasini *et al*., 2014) using the crystal structure of MtUGT71G1 protein (PDB ID: 2ACW chain A; Shao *et al*., 2005) as a template structure. The arrangements of the ligands in the UGT73P12 and BpUGT94B1 proteins were simulated by Autodock Vina 1.1.2 software (Trott and Olson, 2010) with the PyRx 0.8 interface (Dallakyan and Olson, 2015). The maximum size of the grid was selected to obtain reliable results. The results were visualized using PyMOL 1.8 software (Schrödinger, LLC, New York, NY, USA). In addition, the potential rotamers of Arg32 in the UGT73P12 and the minimal distance between Arg32 and UDP‐glucuronic acid were calculated with PyMOL.

## Accession numbers

The nucleotide sequences reported in this paper have been submitted to the DDBJ/GenBank/EBI Data Bank with the following accession numbers: LC314772 (UGT72B31), LC314773 (UGT73B27), LC314778 (UGT73K3), LC314779 (canonical UGT73P12), LC314780 (UGT73P13), LC314783 (UGT87H4), LC314785 (UGT88E21), LC314786 (UGT91H11) and LC315805 (variant UGT73P12).

## Author contributions

KS and TM designed the research; YN, HS, TS, KO, MM, TK, HS and HH performed the research; YN, HS, TS, KO, MM, TK, KT, EO, MH, HS and HH analyzed the data; and YN and HS wrote the paper. YN and HS contributed equally to this work.

## Conflict of interest

The authors declare no conflict of interest.

## Supporting information


**Figure S1.** Overview of the hierarchical clustering of the unigene expression profiles in *G. uralensis*.
**Figure S2.** Proposed biosynthetic pathway for soyasaponins in *G*.* uralensis*.
**Figure S3.** Isolation of recombinant UGT73P12 proteins from *E. coli*.
**Figure S4.** Multiple sequence alignment of candidate UGT proteins in *G*.* uralensis* and their close relatives.
**Figure S5.** UGT73P13 protein can transfer the galactosyl moiety of UDP‐galactose to soyasapogenol B 3‐*O*‐monoglucuronide to produce soyasaponin III.
**Figure S6.** Position and orientation of UDP‐glucose in the MtUGT71G1 protein.
**Figure S7.** Virtual docking of UDP‐sugars onto homology models of UGT73P12 proteins.
**Figure S8.** Inhibitory effect of UDP‐glucose and UDP‐glucuronic acid on the catalytic activity of the canonical UGT73P12 and its R32S mutant protein, respectively (LC‐MS chromatogram related to Figure 8d,e).
**Figure S9.** Enzyme assay of the UGT73B27 and GuUGAT proteins.
**Figure S10.** Comparison of structural models of the canonical UGT73P12 and BpUGT94B1 proteins.
**Figure S11.** Summary of the catalytic functions of the UGT73P12 and UGT73P13 proteins.

**Table S1.** List of PCR primers

**Method S1.** Gene annotation and expression analysis.
**Method S2.** Retrieval of full‐length *UGT* sequences.
**Method S3.** Cloning candidate UGTs.
**Method S4.** Expression and purification of UGT proteins.
**Method S5.** LC‐MS analysis.
