# Supplementary material for: Functional specialization of UDP‐glycosyltransferase 73P12 in licorice to produce a sweet triterpenoid saponin, glycyrrhizin
Source: Plant J. 2019 Jun 26;99(6):1127–43. doi: 10.1111/tpj.14409 (PMC6851746; doi:10.1111/tpj.14409)
Supplement: Supplementary file 1 — Figure S1. Overview of the hierarchical clustering of the unigene expression profiles in G. uralensis. Figure S2. Proposed biosynthetic pathway for soyasaponins in G. uralensis. Figure S3. Isolation of recombinant UGT73P12 proteins from E. coli. Figure S4. Multiple sequence alignment of candidate UGT proteins in G. uralensis and their close relatives. Figure S5. UGT73P13 protein can transfer the galactosyl moiety of UDP‐galactose to soyasapogenol B 3‐O‐monoglucuronide to produce soyasaponin III. Figure S6. Position and orientation of UDP‐glucose in the MtUGT71G1 protein. Figure S7. Virtual docking of UDP‐sugars onto homology models of UGT73P12 proteins. Figure S8. Inhibitory effect of UDP‐glucose and UDP‐glucuronic acid on the catalytic activity of the canonical UGT73P12 and its R32S mutant protein, respectively (LC‐MS chromatogram related to Figure 8d,e). Figure S9. Enzyme assay of the UGT73B27 and GuUGAT proteins. Figure S10. Comparison of structural models of the canonical UGT73P12 and BpUGT94B1 proteins. Figure S11. Summary of the catalytic functions of the UGT73P12 and UGT73P13 proteins. [file TPJ-99-1127-s001.pdf]

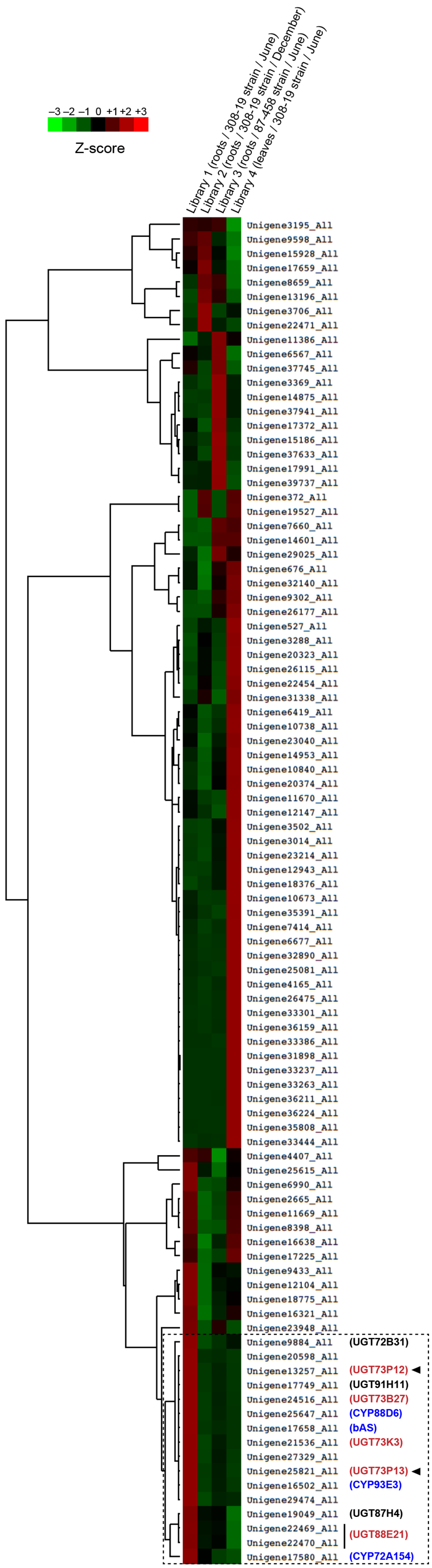
−3 −2 −1 0 +1 +2 +3
Z-score
Library 1 (roots / 308-19 strain / June)
Library 2 (roots / 308-19 strain / December)
Library 3 (roots / 87-458 strain / June)
Library 4 (leaves / 308-19 strain / June)
Unigene3195_All
Unigene9598_All
Unigene15928_All
Unigene17659_All
Unigene8659_All
Unigene13196_All
Unigene3706_All
Unigene22471_All
Unigene11386_All
Unigene6567_All
Unigene37745_All
Unigene3369_All
Unigene14875_All
Unigene37941_All
Unigene17372_All
Unigene15186_All
Unigene37633_All
Unigene17991_All
Unigene39737_All
Unigene372_All
Unigene19527_All
Unigene7660_All
Unigene14601_All
Unigene29025_All
Unigene676_All
Unigene32140_All
Unigene9302_All
Unigene26177_All
Unigene527_All
Unigene3288_All
Unigene20323_All
Unigene26115_All
Unigene22454_All
Unigene31338_All
Unigene6419_All
Unigene10738_All
Unigene23040_All
Unigene14953_All
Unigene10840_All
Unigene20374_All
Unigene11670_All
Unigene12147_All
Unigene3502_All
Unigene3014_All
Unigene23214_All
Unigene12943_All
Unigene18376_All
Unigene10673_All
Unigene35391_All
Unigene7414_All
Unigene6677_All
Unigene32890_All
Unigene25081_All
Unigene4165_All
Unigene26475_All
Unigene33301_All
Unigene36159_All
Unigene33386_All
Unigene31898_All
Unigene33237_All
Unigene33263_All
Unigene36211_All
Unigene36224_All
Unigene35808_All
Unigene33444_All
Unigene4407_All
Unigene25615_All
Unigene6990_All
Unigene2665_All
Unigene11669_All
Unigene8398_All
Unigene16638_All
Unigene17225_All
Unigene9433_All
Unigene12104_All
Unigene18775_All
Unigene16321_All
Unigene23948_All
Unigene9884_All (UGT72B31)
Unigene20598_All
Unigene13257_All (UGT73P12)
Unigene17749_All (UGT91H11)
Unigene24516_All (UGT73B27)
Unigene25647_All (CYP88D6)
Unigene17658_All (bAS)
Unigene21536_All (UGT73K3)
Unigene27329_All
Unigene25821_All (UGT73P13)
Unigene16502_All (CYP93E3)
Unigene29474_All
Unigene19049_All (UGT87H4)
Unigene22469_All
Unigene22470_All
(UGT88E21)
Unigene17580_All (CYP72A154)

Figure S1

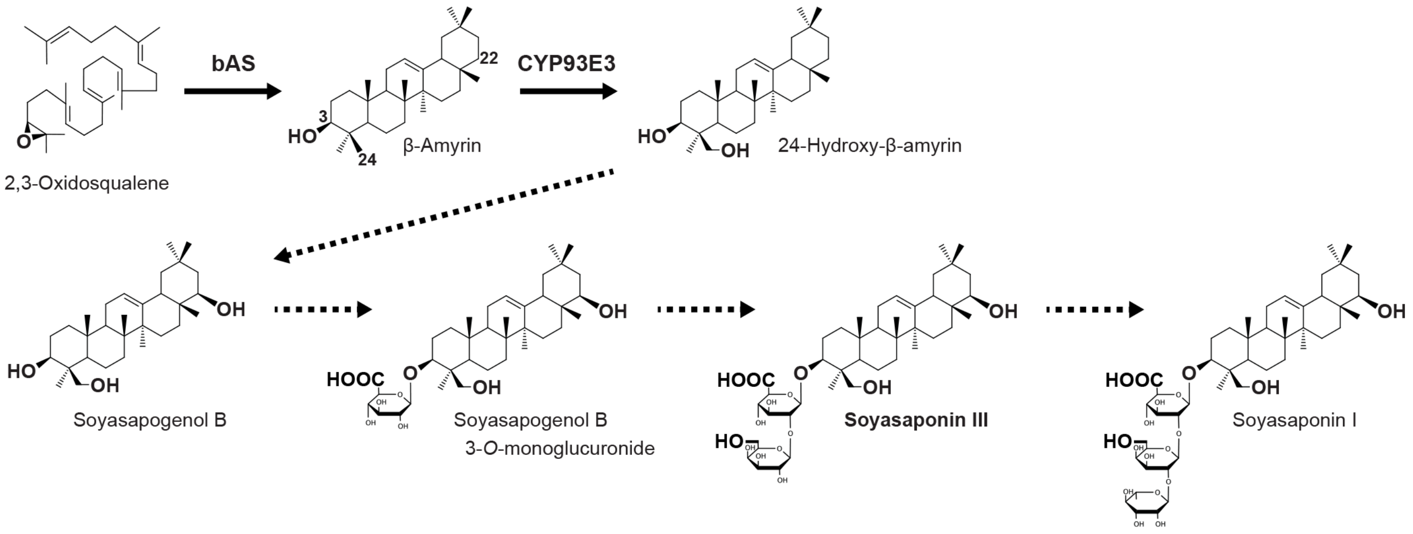
bAS
CYP93E3
2,3-Oxidosqualene
β-Amyrin
24-Hydroxy-β-amyrin
Soyasapogenol B
Soyasapogenol B
3-O-monoglucuronide
Soyasaponin III
Soyasaponin I

Figure S2

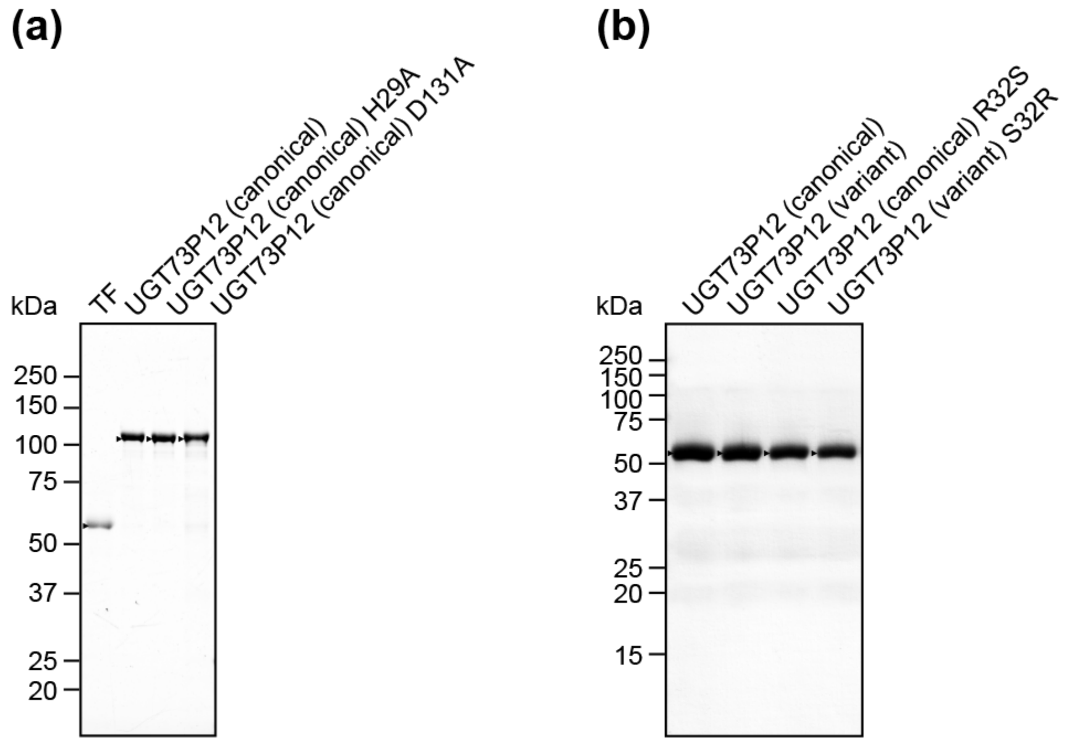
(a)
kDa
TF
UGT73P12 (canonical)
UGT73P12 (canonical) H29A
UGT73P12 (canonical) D131A
250
150
100
75
50
37
25
20
(b)
kDa
UGT73P12 (canonical)
UGT73P12 (variant)
UGT73P12 (canonical) R32S
UGT73P12 (variant) S32R
250
150
100
75
50
37
25
20
15

Figure S3

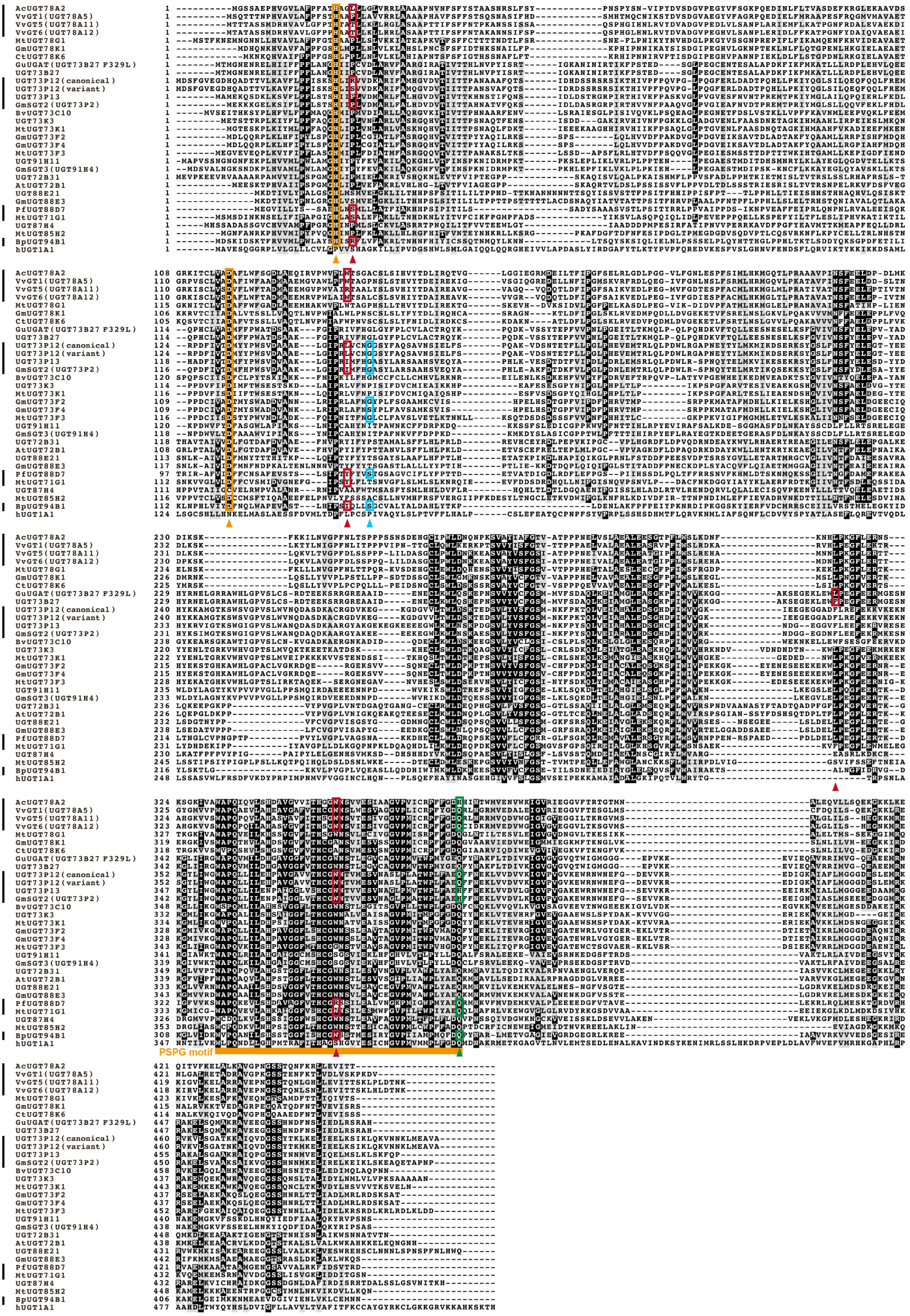


Figure S4

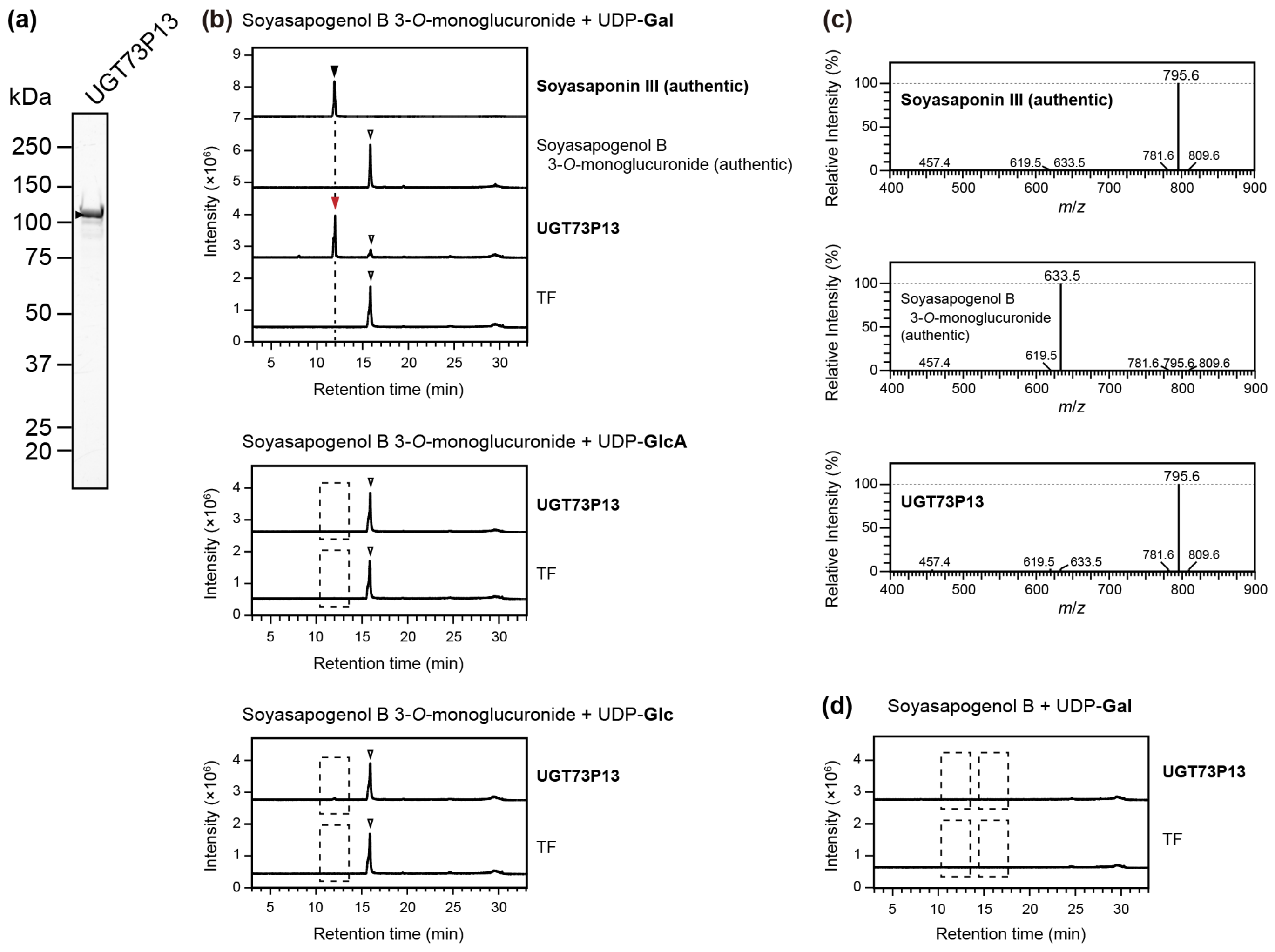
(a)
UGT73P13
kDa
250
150
100
75
50
37
25
20
(b) Soyasapogenol B 3-O-monoglucuronide + UDP-Gal
Soyasaponin III (authentic)
Soyasapogenol B 3-O-monoglucuronide (authentic)
UGT73P13
TF
Intensity (×10⁶)
Retention time (min)
Soyasapogenol B 3-O-monoglucuronide + UDP-GlcA
UGT73P13
TF
Soyasapogenol B 3-O-monoglucuronide + UDP-Glc
UGT73P13
TF
(c)
Relative Intensity (%)
Soyasaponin III (authentic)
795.6
457.4
619.5
633.5
781.6
809.6
m/z
Soyasapogenol B 3-O-monoglucuronide (authentic)
633.5
457.4
619.5
781.6
795.6
809.6
UGT73P13
795.6
457.4
619.5
633.5
781.6
809.6
(d) Soyasapogenol B + UDP-Gal
UGT73P13
TF
Retention time (min)

Figure S5

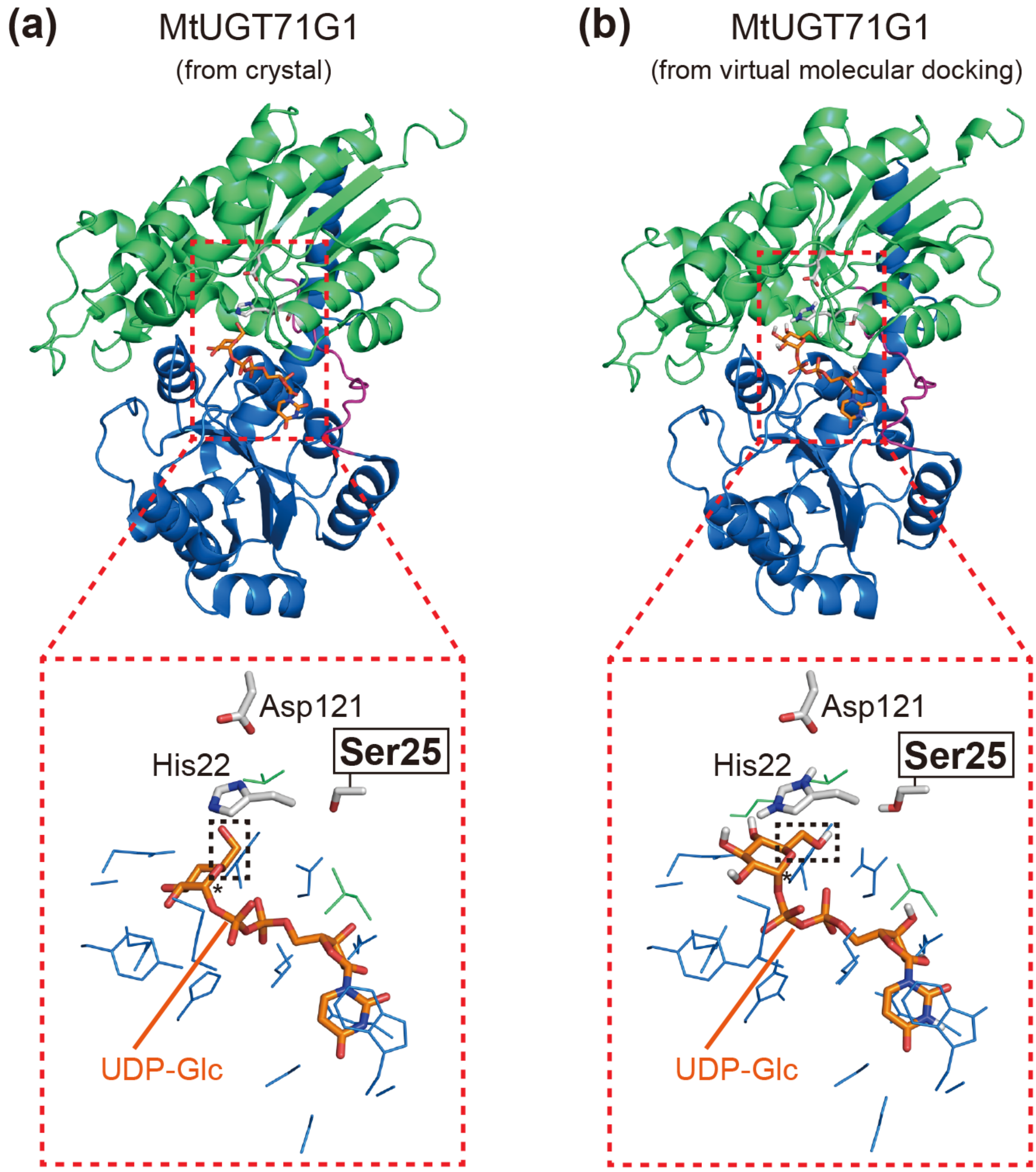
(a)
MtUGT71G1
(from crystal)
Asp121
His22
Ser25
*
UDP-Glc
(b)
MtUGT71G1
(from virtual molecular docking)
Asp121
His22
Ser25
*
UDP-Glc

Figure S6

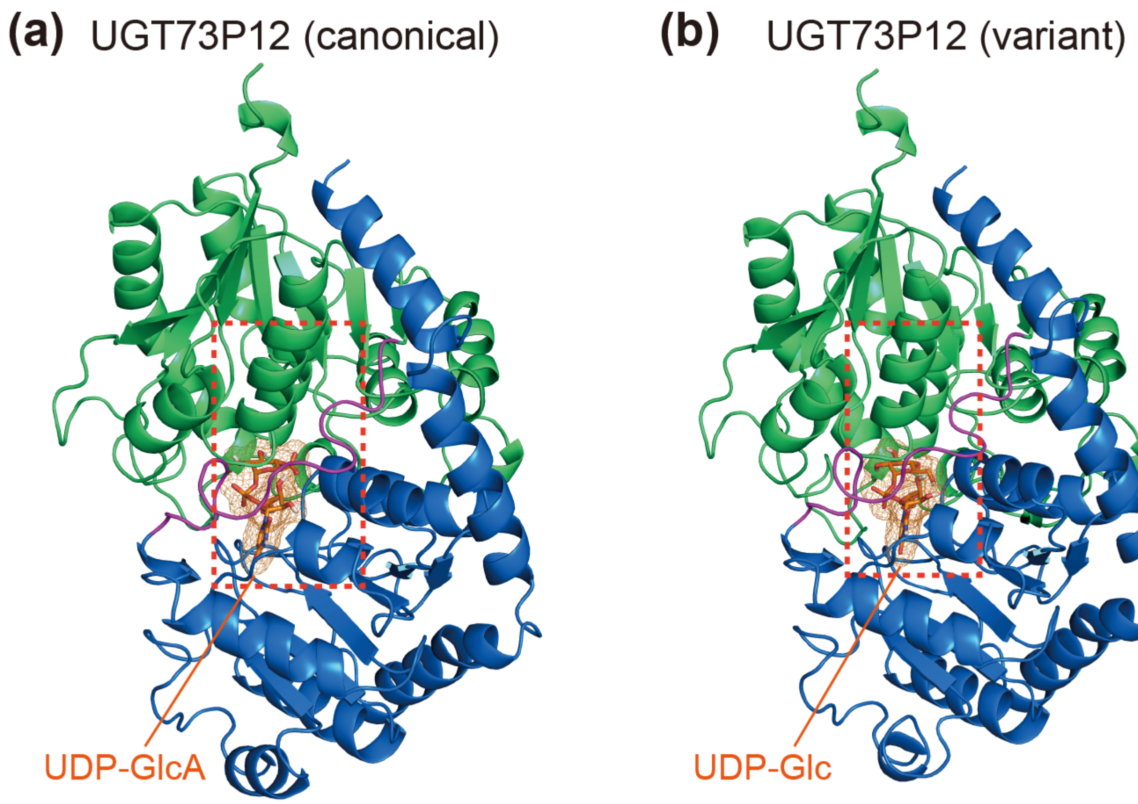


Figure S7

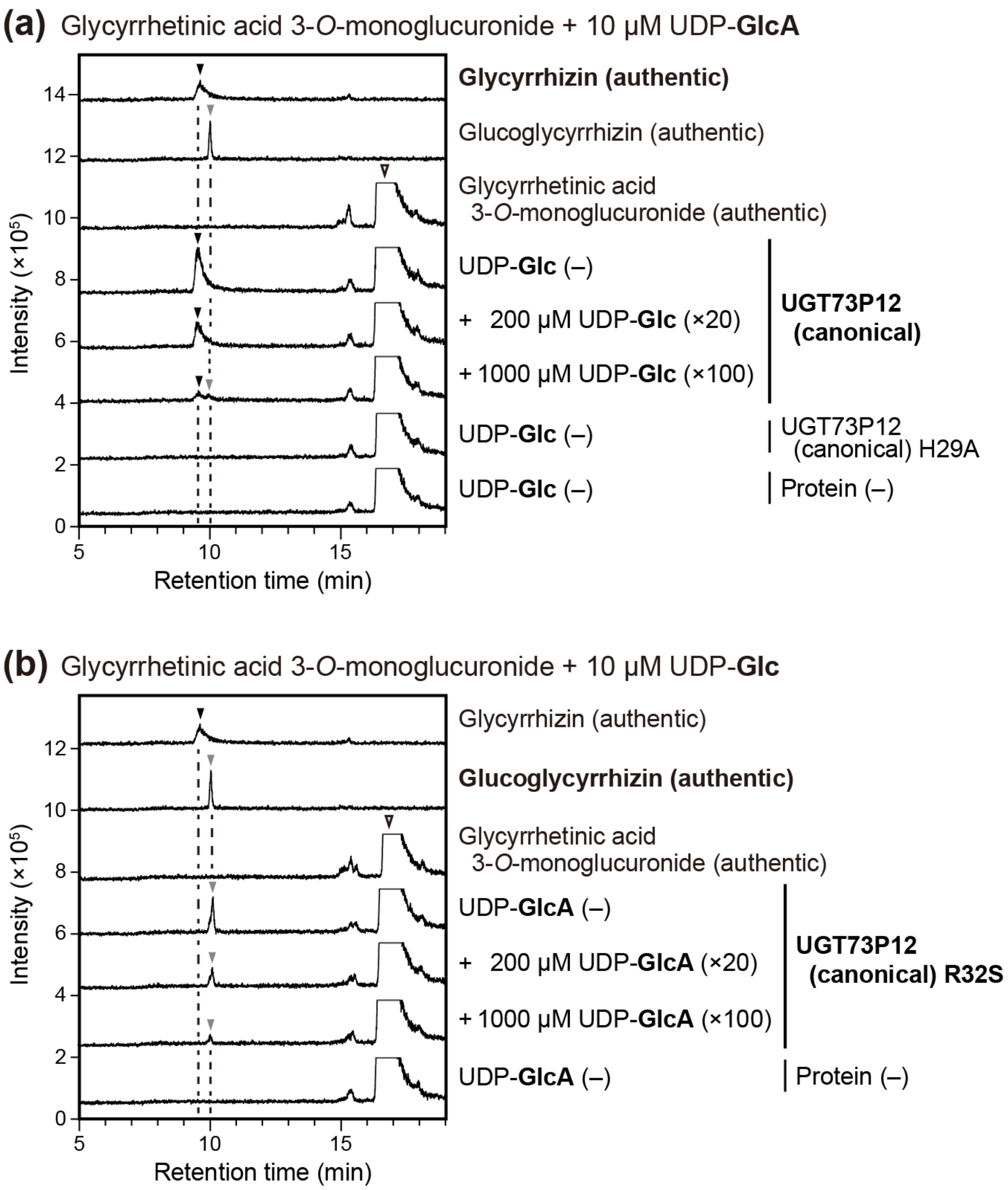
(a) Glycyrrhetinic acid 3-O-monoglucuronide + 10 μM UDP-GlcA
Glycyrrhizin (authentic)
Glucoglycyrrhizin (authentic)
Glycyrrhetinic acid 3-O-monoglucuronide (authentic)
UDP-Glc (–)
+ 200 μM UDP-Glc (×20)
+ 1000 μM UDP-Glc (×100)
UGT73P12 (canonical)
UDP-Glc (–)
UGT73P12 (canonical) H29A
UDP-Glc (–)
Protein (–)
Intensity (×10^5)
Retention time (min)
(b) Glycyrrhetinic acid 3-O-monoglucuronide + 10 μM UDP-Glc
Glycyrrhizin (authentic)
Glucoglycyrrhizin (authentic)
Glycyrrhetinic acid 3-O-monoglucuronide (authentic)
UDP-GlcA (–)
+ 200 μM UDP-GlcA (×20)
+ 1000 μM UDP-GlcA (×100)
UGT73P12 (canonical) R32S
UDP-GlcA (–)
Protein (–)
Intensity (×10^5)
Retention time (min)

Figure S8

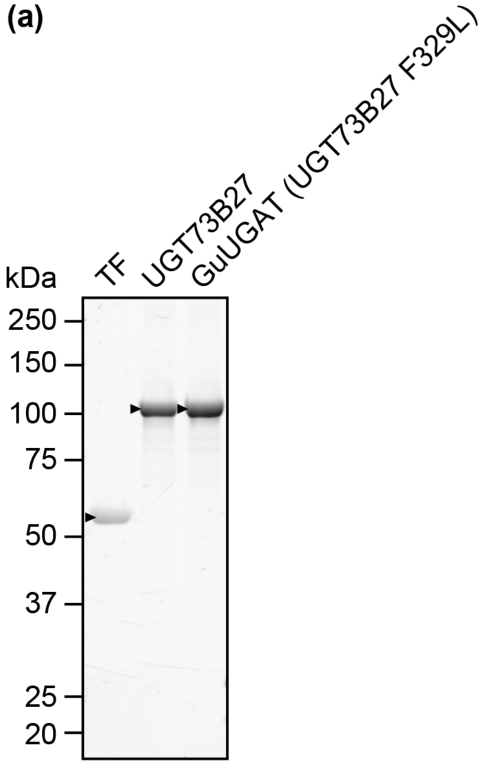
(a)
kDa
TF
UGT73B27
GuUGAT (UGT73B27 F329L)
250
150
100
75
50
37
25
20

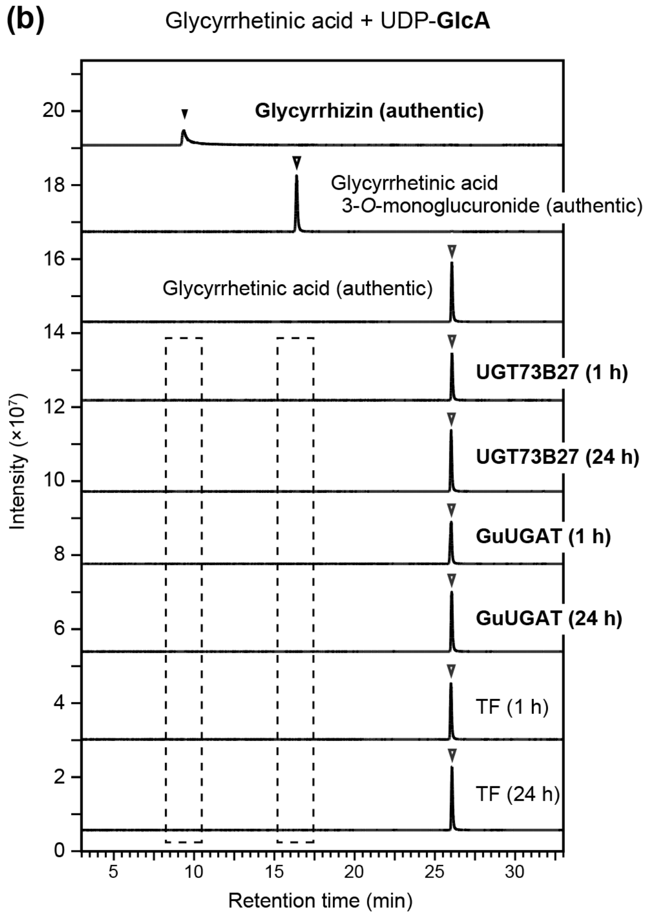
(b)
Glycyrrhetinic acid + UDP-**GlcA**
Glycyrrhizin (authentic)
Glycyrrhetinic acid
3-O-monoglucuronide (authentic)
Glycyrrhetinic acid (authentic)
UGT73B27 (1 h)
UGT73B27 (24 h)
GuUGAT (1 h)
GuUGAT (24 h)
TF (1 h)
TF (24 h)
Intensity (×10^7)
Retention time (min)

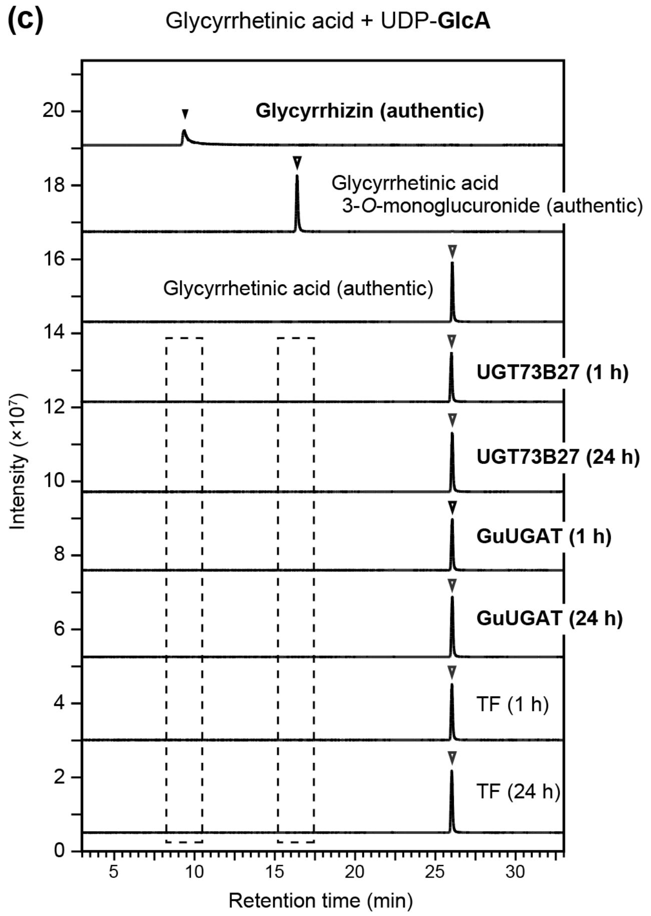
(c)
Glycyrrhetinic acid + UDP-**GlcA**
Glycyrrhizin (authentic)
Glycyrrhetinic acid
3-O-monoglucuronide (authentic)
Glycyrrhetinic acid (authentic)
UGT73B27 (1 h)
UGT73B27 (24 h)
GuUGAT (1 h)
GuUGAT (24 h)
TF (1 h)
TF (24 h)
Intensity (×10^7)
Retention time (min)

Figure S9

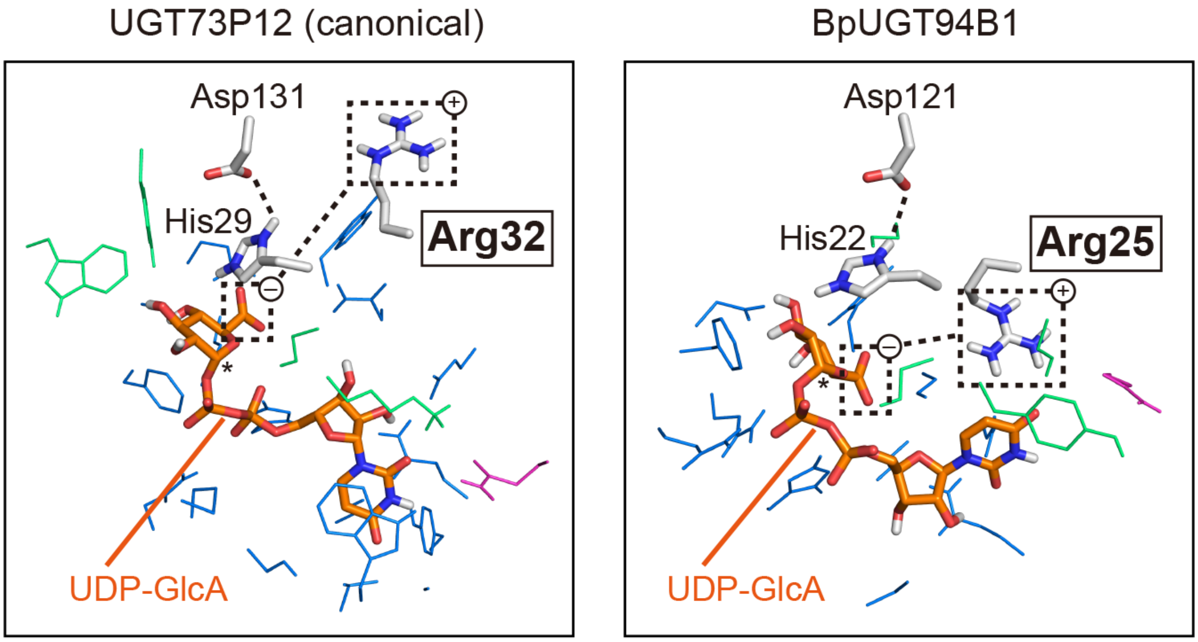

UGT73P12 (canonical)
Asp131
His29
Arg32
UDP-GlcA
BpUGT94B1
Asp121
His22
Arg25
UDP-GlcA


Figure S10

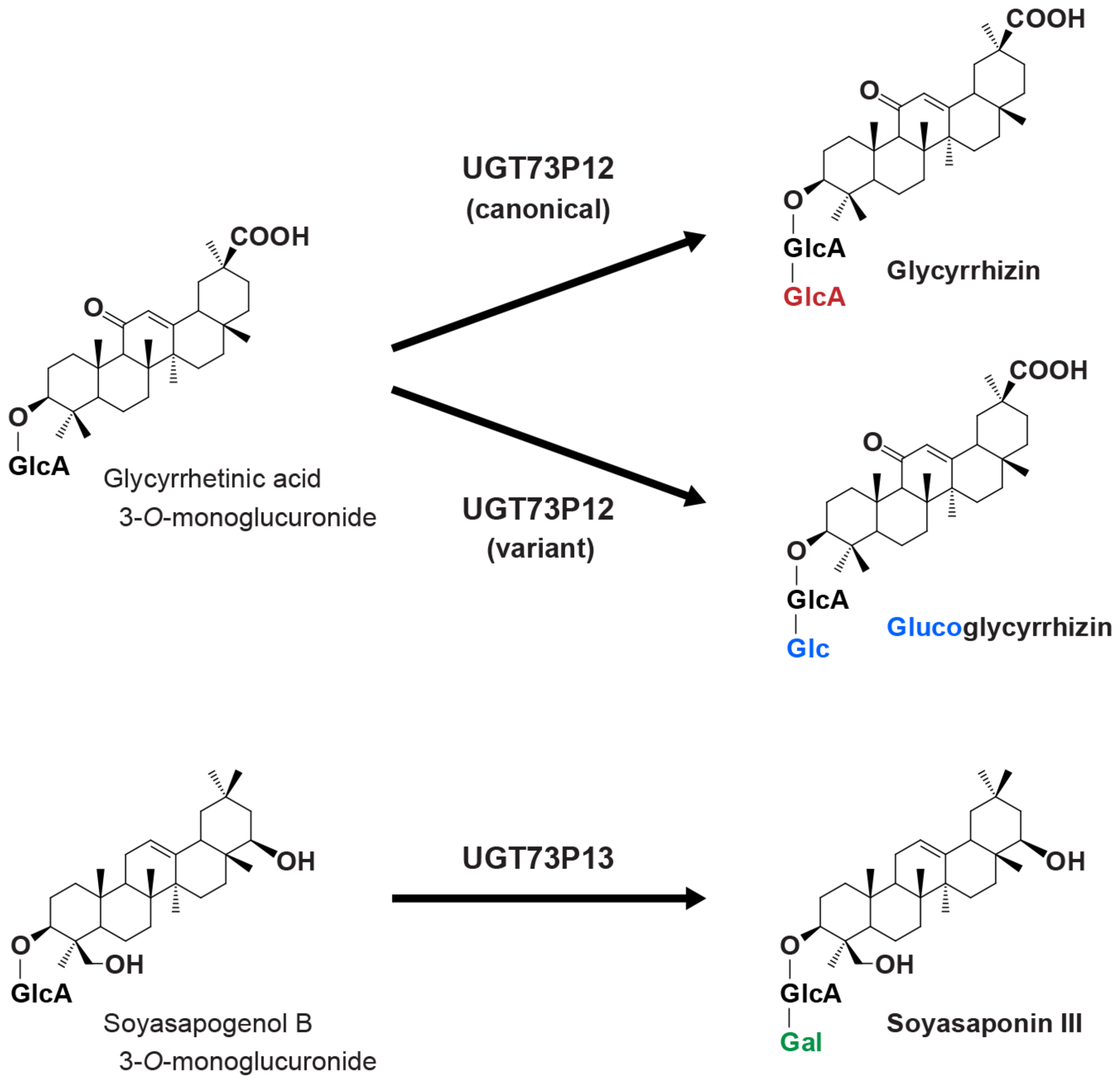
COOH
O
O
GlcA
Glycyrrhetinic acid
3-O-monoglucuronide
UGT73P12
(canonical)
UGT73P12
(variant)
O
GlcA
GlcA
Glycyrrhizin
O
GlcA
Glc
Glucoglycyrrhizin
OH
OH
O
GlcA
Soyasapogenol B
3-O-monoglucuronide
UGT73P13
OH
OH
O
GlcA
Gal
Soyasaponin III

Figure S11
