# Supplementary material for: Functional specialization of UDP‐glycosyltransferase 73P12 in licorice to produce a sweet triterpenoid saponin, glycyrrhizin
Source: Plant J. 2019 Jun 26;99(6):1127–43. doi: 10.1111/tpj.14409 (PMC6851746; doi:10.1111/tpj.14409)
Supplement: Supplementary file 2 — Table S1. List of PCR primers [file TPJ-99-1127-s002.pdf]

**Table S1.** List of PCR primers

| No | Primer name | Primer sequence (5' to 3') |
|---|---|---|
| 1 | UGT72B31-forward | caccatggaagtcccaaaggaagaagtga |
| 2 | UGT72B31-reverse | ttaatttgttacagtggcattgttgc |
| 3 | UGT73B27-forward | caccatgaccatgggtaacgagaatcggga |
| 4 | UGT73B27-reverse | ttaatgggcacgcgacctcaaatcct |
| 5 | UGT73K3-forward | caccatggaaacaagtacaacccggccgtt |
| 6 | UGT73K3-reverse | ccgagttttgattgctcccaatgga |
| 7 | UGT73P12-forward | caccatggactcctttggggttgaaggtga |
| 8 | UGT73P12-reverse | ttaagccactgcctccattaatttgt |
| 9 | UGT73P13-forward | caccatggccatggagaagcaaagtgata |
| 10 | UGT73P13-reverse | cggttgaaacttggaaagtttgagggac |
| 11 | UGT87H4-forward | caccatgcatagctccgccgacgcct |
| 12 | UGT87H4-reverse | tcaatgcttagttatgttaactgagcc |
| 13 | UGT88E21-forward | caccatgaaagacaccatagttctatacc |
| 14 | UGT88E21-reverse | ttattgccagtgaagattgaatggg |
| 15 | UGT91H11-forward | caccatggctccagtttcatctaatggca |
| 16 | UGT91H11-reverse | ttagctgttggaaggaaccctatac |
| 17 | UGT73P12-upstream-forward | cctgatttcccaacactagactctg |
| 18 | UGT73P12-downstream-reverse | caattcacgatattttgatgatggac |
| 19 | UGT73P12-H29A-forward | cttcatctcgaaaagtgctctcatccgtgtggtgg |
| 20 | UGT73P12-H29A-reverse | ccaccacacggatgagagcacttttcgagatgaag |
| 21 | UGT73P12-D131A-forward | gatttcatagtcactgccatgtactacccttggag |
| 22 | UGT73P12-D131A-reverse | ctccaagggtagtacatggcagtgactatgaaatc |
| 23 | UGT73P12 (canonical)-R32S-forward | gaaaagtcatctcatcagtgtggtggacaaagcaaggatcttcg |
| 24 | UGT73P12 (canonical)-R32S-reverse | gctttgtccaccacactgatgagatgacttttcgagatgaag |
| 25 | UGT73P12 (variant)-S32R-forward | gtcatctcatccgtgtggtggacaaagcaaggatcttc |
| 26 | UGT73P12 (variant)-S32R-reverse | ctttgtccaccacacggatgagatgactttttgagatgaag |
