# Supplementary material for: Functional specialization of UDP‐glycosyltransferase 73P12 in licorice to produce a sweet triterpenoid saponin, glycyrrhizin
Source: Plant J. 2019 Jun 26;99(6):1127–43. doi: 10.1111/tpj.14409 (PMC6851746; doi:10.1111/tpj.14409)
Supplement: Supplementary file 3 — Method S1. Gene annotation and expression analysis. Method S2. Retrieval of full‐length UGT sequences. Method S3. Cloning candidate UGTs. Method S4. Expression and purification of UGT proteins. Method S5. LC‐MS analysis. [file TPJ-99-1127-s003.pdf]

SUPPLEMENTARY METHODS

Method S1

Gene annotation and expression analysis

A dataset of non-redundant unigene sequences and their expression profiles was obtained via RNA-Seq of *G. uralensis* cDNA libraries (Ramilowski *et al.*, 2013). Putative *UGTs* were annotated as previously established (Ramilowski *et al.*, 2013; Mochida *et al.*, 2017), with the following modified procedure. Possible open reading frames within the unigene sequences were predicted by TransDecoder 2.0.1 software (Haas *et al.*, 2013), and were searched for *UGTs* using a hidden Markov model-based profile of the UGT domain (Pfam family: PF00201 in the Pfam database 30.0; Sonnhammer *et al.*, 1998) as a query with HMMER 3.1b2 software (Eddy, 2009). Unigenes that were found to encode the UGT domain were annotated as putative *UGTs*. The unigene expression levels were previously calculated in fragments per kilobase per million reads (FPKM) units (Ramilowski *et al.*, 2013). The FPKM values were normalized as Z-score values among the libraries, and hierarchical clustering of the Z-score values was performed with Cluster 3.0 software (de Hoon *et al.*, 2004). The results were then illustrated using Java TreeView 1.1.6r2 software (Saldanha, 2004).

Method S2

Retrieval of full-length *UGT* sequences

Full-length coding sequences of the eight candidate *UGTs* (*UGT72B31*, *UGT73B27*, *UGT73K3*, *UGT73P12*, *UGT73P13*, *UGT87H4*, *UGT88E21*, and *UGT91H11*) were retrieved from 12 unigene sequences, as follows. The unigene sequences were first aligned to evaluate whether each unigene contained a full-length coding sequence. From this analysis, six full-length coding sequences were retrieved from Unigene9884_All (*UGT72B31*), Unigene13257_All (*UGT73P12*), Unigene17749_All (*UGT91H11*), Unigene24516_All (*UGT73B27*), Unigene21536_All (*UGT73K3*), and Unigene25821_All (*UGT73P13*). Although the other six unigenes contained partial coding sequences, the corresponding sequences of three unigenes (Unigene19049_All and Unigene22469_All/Unigene22470_All) were also found in expressed sequence tags (ESTs) derived from a cDNA library of licorice stolons (Sudo *et al.*, 2009), and were found to encode two full-length UGT proteins (Unigene22469_All and

Unigene22470_All were found to be derived from a single gene by comparing their cloned cDNA sequences). Therefore, we were able to retrieve *UGT87H4* and *UGT88E21* from Unigene19049_All and Unigene22469_All/Unigene22470_All, respectively.

Method S3

Cloning candidate *UGTs*

RNA was extracted and cDNA prepared as previously described (Seki *et al.*, 2008), with minor modifications. Total RNA was first extracted from the roots or stolons of three *G. uralensis* strains: 308-19 strain (Ramilowski *et al.*, 2013), a glycyrrhizin-producing strain called Hokkaido-iryodai (Sudo *et al.*, 2009), and 83-555 strain (Hayashi *et al.*, 2013). The RNAs were subjected to reverse transcription for cDNA preparation using the SMART RACE cDNA Amplification Kit (Clontech, Mountain View, CA, USA). The full-length coding regions encoding *UGT72B31*, *UGT73B27*, *UGT73K3*, *UGT73P12*, *UGT87H4*, *UGT88E21*, and *UGT91H11* were amplified by PCR from the cDNA of the Hokkaido-iryodai strain with the gene-specific primers 1-8 and 11-16. Table S1 lists the PCR primers. The coding region of *UGT73P13* was amplified by PCR from the cDNA of the 308-19 strain with primers 9 and 10. The coding region of a variant *UGT73P12* was amplified by PCR from the cDNA of the 83-555 strain with primers 7 and 8, after its sequence had been determined with primers 17 and 18. These PCR products were cloned directly into a pENTR/D-TOPO entry vector (Invitrogen, Carlsbad, CA, USA) for sequence determination.

Method S4

Expression and purification of UGT proteins

The expression vectors were introduced into *E. coli* Rosetta 2(DE3)pLysS competent cells (Novagen, Madison, WI, USA). The *E. coli* transformants were grown at 37°C in LB medium containing 50 mg/L carbenicillin and 30 mg/L chloramphenicol until the cultures had achieved an optical density of 0.5 at 600 nm. The cultures were then supplemented with isopropyl *β*-D-thiogalactopyranoside at a final concentration of 500 μM and incubated for an additional 24 h at 15°C. The cells were harvested by centrifugation and disrupted by ultrasonic treatment in a solution containing 50 mM sodium phosphate buffer (pH 7.0), 500 mM NaCl, and 20% glycerol. The cell extracts

were then centrifuged at 20,400 × *g* for 20 min at 4°C, and the resulting supernatants were applied to TALON metal affinity resin (Clontech). The resin was washed with cell disruption solution containing 18 mM imidazole, and proteins on the resin were then eluted with the same solution containing 180 mM imidazole. These purified proteins were used for the initial characterization of the canonical UGT73P12 protein (WT, H29A, and D131A) and UGT73P13 protein. For detailed characterization of the canonical UGT73P12 (WT and R32S) and variant UGT73P12 (WT and S32R) proteins with respect to their sugar donor selectivity and kinetic parameters, a trigger factor tag fused to the UGT73P12 protein was cleaved using the Factor Xa Cleavage Capture Kit (Novagen) and removed by passage through a His GraviTrap column (GE Healthcare, Chalfont St. Giles, UK). Alternatively, His-tagged HRV 3C protease (TaKaRa Bio, Shiga, Japan) was also used to cleave the trigger factor tag from the canonical UGT73P12 protein (WT, H29A, and R32S). The concentrations of these purified proteins were determined using a protein assay kit (Bio-Rad, Richmond, CA, USA) with bovine serum albumin (BSA) as the standard.

Method S5

LC-MS analysis

The samples were applied to an ACQUITY UPLC HSS C18 column (2.1 × 150 mm column and 2.1 × 5 mm VanGuard pre-column; particle size: 1.8 µm; Waters, Milford, MA, USA) for LC-MS analysis with an ACQUITY UPLC/MS system (Waters). Two types of solvent were used in the column: solvent 1 consisted of water containing 0.025% (v/v) acetic acid and solvent 2 consisted of acetonitrile containing 0.025% (v/v) acetic acid. The column was kept at 30°C, and a mixture of solvents 1 and 2 (70:30 v/v) was applied to the column at a flow rate of 0.20 mL/min for 6 min. Elution was then performed by increasing the proportion of solvent 2 from 30% to 100% over 22 min (40% at 6 min, 50% at 18 min, and 100% at 28 min). The last condition was then maintained for 3.5 min. An ACQUITY TQ Detector (Waters) was used to detect reaction products in electrospray ionization (ESI) negative ion mode with selected ion monitoring (*m/z* 469.3, 631.4, 645.4, 777.5, 793.5, 807.5, and 821.4 for the UGT73P12-mediated reactions, and *m/z* 457.4, 619.5, 633.5, 781.6, 795.6, and 809.6 for the UGT73P13-mediated reactions). The other parameters were set as follows: capillary voltage 2.5 kV, cone voltage 80 V, extractor voltage 3 V, source temperature 150°C,

desolvation temperature 350°C, cone gas flow 50 L/h, and desolvation gas flow 600 L/h. The amount of reaction products was determined as the peak area using MassLynx software (Waters). Kinetic parameters were calculated by fitting a Michaelis–Menten curve to the raw kinetic data using GraphPad Prism 6.0h software (GraphPad Software, San Diego, CA, USA). The raw data were calibrated beforehand with standard curves of authentic glycyrrhizin and glucoglycyrrhizin.

SUPPLEMENTARY REFERENCES

de Hoon, M.J., Imoto, S., Nolan, J. and Miyano, S. (2004) Open source clustering software. *Bioinformatics*, **20**, 1453-1454. https://doi.org/10.1093/bioinformatics/bth078

Eddy, S.R. (2009) A new generation of homology search tools based on probabilistic inference. *Genome inform.*, **23**, 205-211. https://doi.org/10.1142/9781848165632_0019

Haas, B.J., Papanicolaou, A., Yassour, M., Grabherr, M., Blood, P.D., Bowden, J., Couger, M.B., Eccles, D., Li, B., Lieber, M., MacManes, M.D., Ott, M., Orvis, J., Pochet, N., Strozzi, F., Weeks, N., Westerman, R., William, T., Dewey, C.N., Henschel, R., LeDuc, R.D., Friedman, N. and Regev, A. (2013) *De novo* transcript sequence reconstruction from RNA-seq using the Trinity platform for reference generation and analysis. *Nature protoc.*, **8**, 1494-1512. https://doi.org/10.1038/nprot.2013.084

Hayashi, H., Fujii, I., Iinuma, M., Shibano, M., Ozaki, K. and Watanabe, H. (2013) Characterization of a glycyrrhizin-deficient strain of *Glycyrrhiza uralensis*. *Biol. Pharm. Bull.*, **36**, 1448-1453. https://doi.org/10.1248/bpb.b13-00164

Mochida, K., Sakurai, T., Seki, H., Yoshida, T., Takahagi, K., Sawai, S., Uchiyama, H., Muranaka, T. and Saito, K. (2017) Draft genome assembly and annotation of *Glycyrrhiza uralensis*, a medicinal legume. *Plant J.*, **89**, 181-194. https://doi.org/10.1111/tpj.13385

Ramilowski, J.A., Sawai, S., Seki, H., Mochida, K., Yoshida, T., Sakurai, T., Muranaka, T., Saito, K. and Daub, C.O. (2013) *Glycyrrhiza uralensis* transcriptome landscape and study of phytochemicals. *Plant Cell Physiol.*, **54**, 697-710. https://doi.org/10.1093/pcp/pct057

Saldanha, A.J. (2004) Java Treeview - extensible visualization of microarray data. *Bioinformatics*, **20**, 3246-3248. https://doi.org/10.1093/bioinformatics/bth349

Seki, H., Ohyama, K., Sawai, S., Mizutani, M., Ohnishi, T., Sudo, H., Akashi, T., Aoki, T., Saito, K. and Muranaka, T. (2008) Licorice β-amyrin 11-oxidase, a cytochrome P450 with a key role in the biosynthesis of the triterpene sweetener glycyrrhizin. *Proc. Natl. Acad. Sci. U. S. A.*, **105**, 14204-14209. https://doi.org/10.1073/pnas.0803876105

Sonnhammer, E.L., Eddy, S.R., Birney, E., Bateman, A. and Durbin, R. (1998) Pfam: multiple sequence alignments and HMM-profiles of protein domains. *Nucleic Acids Res.*, **26**, 320-322. https://doi.org/10.1093/nar/26.1.320

Sudo, H., Seki, H., Sakurai, N., Suzuki, H., Shibata, D., Toyoda, A., Totoki, Y., Sakaki, Y., Iida, O., Shibata, T., Kojoma, M., Muranaka, T. and Saito, K. (2009) Expressed sequence tags from rhizomes of *Glycyrrhiza uralensis*. *Plant Biotech.*, **26**, 105-107. https://doi.org/10.5511/plantbiotechnology.26.105
