# Supplementary material for: Functional specialization of UDP‐glycosyltransferase 73P12 in licorice to produce a sweet triterpenoid saponin, glycyrrhizin
Source: Plant J. 2019 Jun 26;99(6):1127–43. doi: 10.1111/tpj.14409 (PMC6851746; doi:10.1111/tpj.14409)
Supplement: Supplementary file 4 [file TPJ-99-1127-s004.docx]

**Supplementary Figure legends**

**Figure S1.** Overview of the hierarchical clustering of the unigene expression profiles in *G. uralensis*.

The heatmap shows the relative expression levels of the unigenes in the four libraries. The color scale (–3.00 to 3.00 from green to red, respectively) represents the Z-score, which corresponds to the relative expression level. The part of the heatmap inside the dotted box indicates unigenes with expression patterns that were highly correlated with those of other glycyrrhizin and/or soyasaponin biosynthetic genes, *bAS*, *CYP88D6*, *CYP72A154*, and *CYP93E3* (blue). The bar spanning two unigenes indicates that the unigenes are derived from one gene. The dendrogram along the left side of the heat map indicates the hierarchical clustering of unigenes. The *UGTs* shown in black or red were named in this study, and those shown in red were further used to screen for functional proteins. Arrowheads indicate the UGTs characterized in this study.

**Figure S2.** Proposed biosynthetic pathway for soyasaponins in *G*. *uralensis*.

The following terms are used: bAS, β-amyrin synthase; and CYP93E3, β-amyrin 24-oxidase. The position of carbon atoms related to this pathway is indicated on the chemical structure of β-amyrin. Broken arrows indicate unidentified and expected routes to produce the soyasaponins in *G*. *uralensis*.

**Figure S3.** Isolation of recombinant UGT73P12 proteins from *E. coli*.

(a) SDS-PAGE analysis of isolated trigger factor (TF)-tagged recombinant proteins of the canonical UGT73P12 and its two alanine-scanning mutants (H29A and D131A). The TF protein was prepared as a negative control. Stain-free gels (Bio-Rad) were used to visualize the UGT bands. The ultraviolet-induced fluorescent signals were detected by the GelDoc XR+ Imaging System (Bio-Rad), and the positions of molecular standards are indicated. *Arrowheads* indicate the UGT bands.

(b) SDS-PAGE analysis of purified recombinant proteins, canonical UGT73P12, variant UGT73P12, canonical UGT73P12 R32S, and variant UGT73P12 S32R proteins, from which the TF tag had been removed. The gel was stained with Coomassie Brilliant Blue, and the positions of molecular standards are indicated. *Arrowheads* indicate the UGT bands.

**Figure S4.** Multiple sequence alignment of candidate UGT proteins in *G*. *uralensis* and their close relatives.

The species names of the close relatives that were previously characterized in other plants are abbreviated as follows: Ac, *Aralia cordata*; Vv, *Vitis vinifera*; Mt, *Medicago truncatula*; Gm, *Glycine max*; Ct, *Clitoria ternatea*; Bv, *Barbarea vulgaris*; At, *Arabidopsis thaliana*; Pf, *Perilla frutescens*; and Bp, *Bellis perennis*. The human (h) UGT1A1 was used as the outgroup. The catalytically essential histidine and aspartate residues are shown in the orange box with orange *arrowheads* and the PSPG motif sequence is underlined in orange. The positions of the amino acid residues that define the UDP-sugar selectivity are indicated by red, blue, or green *arrowheads*. Within the positions, the typical differences in the amino acid residues are further highlighted by red, blue, or green boxes. The arginine residues in the red boxes are the essential residues of UGT proteins that provide high specificity for UDP-glucuronic acid. The serine residues in the blue boxes represent important residues that increase UDP-sugar specificity in UGTs, including a serine residue (Ser127) in PfUGT88D7 that increases the specificity for UDP-glucuronic acid (Noguchi *et al.*, 2009) and a serine residue (Ser138) in GmUGT73F4 that is necessary for its strict UDP-xylose specificity (Sayama *et al.*, 2012). The histidine residues in the green boxes are important residues that restrict UDP-sugar selectivity for UDP-galactose specificity in a flavonoid UGT: *A. cordata* UDP-galactose:anthocyanin galactosyltransferase (UGT78A2) conserves a histidine residue (His374) at the end of the PSPG motif, and replacement of this histidine residue with a glutamine residue confers glucosyltransferase activity on this protein (Kubo *et al.*, 2004). The corresponding histidine residues of GmSGT2 and UGT73P13 proteins (His392 and His397, respectively) are also inside the green box.

**Figure S5.** UGT73P13 protein can transfer the galactosyl moiety of UDP-galactose to soyasapogenol B 3-*O*-monoglucuronide to produce soyasaponin III.

(a) SDS-PAGE analysis of purified trigger factor (TF)-tagged recombinant UGT73P13 protein. Stain-free gels (Bio-Rad) were used to visualize the UGT bands. The ultraviolet-induced fluorescent signals were detected with the GelDoc XR+ Imaging System (Bio-Rad), and the positions of molecular standards are indicated. *Arrowheads* indicate the UGT bands.

(b) LC-MS chromatogram of authentic compounds (soyasapogenol B 3-*O*-monoglucuronide and soyasaponin III) and *in vitro* reaction products. The enzyme reaction of the purified UGT73P13 protein was performed for 1 h in the presence of soyasapogenol B 3-*O*-monoglucuronide as the sugar acceptor substrate and UDP-sugars, including UDP-galactose, UDP-glucuronic acid, and UDP-glucose, as described in the EXPERIMENTAL PROCEDURES. The reaction with TF protein was used as a negative control. The following abbreviations are used: UDP-Gal, UDP-galactose; UDP-GlcA, UDP-glucuronic acid; and UDP-Glc, UDP-glucose. The red *arrowhead* indicates the reaction product of UGT73P13 protein, which matches authentic soyasaponin III in terms of retention time (12.0 min) and the exact mass of the deprotonated ion [M-H]^–^ (*m/z* = 795.6).

(c) MS spectra of authentic compounds (soyasapogenol B 3-*O*-monoglucuronide and soyasaponin III) and the reaction product detected in (b) as the UGT73P13 activity.

(d) LC-MS analysis of reaction mixtures. The enzyme reactions of the purified proteins, UGT73P13 and TF (negative control), were performed with the same reaction conditions as (b) except that soyasapogenol B was used as the sugar acceptor substrate. UDP-galactose is abbreviated as UDP-Gal.

Broken boxes in (b) and (d) indicate that no definite reaction product was detected. The LC-MS data in (b)–(d) represent three separate reactions performed with the same preparation of each purified protein.

**Figure S6.** Position and orientation of UDP-glucose in the MtUGT71G1 protein.

(a) The crystal data of MtUGT71G1 protein bound to UDP-glucose (PDB ID: 2ACW chain A). The hydrogen atom coordinates are missing because of the insufficient resolution of the structural data.

(b) Simulated position of UDP-glucose in the MtUGT71G1 protein. Virtual docking of UDP-glucose (orange) onto the crystal structure of MtUGT71G1 protein (PDB ID: 2ACW chain A) was performed, after three-dimensional coordinate data related to UDP-glucose had been removed from the crystal data.

The N- and C-domains, and their linker region are shown in green, blue, and magenta, respectively. The position of the sugar donor pocket in the MtUGT71G1 protein is shown in the orange dotted box with the catalytically essential residues His22 and Asp121. Ser25, which is in the same position as Arg32 of the canonical UGT73P12 in the alignment of their protein sequences, is also shown. The close-up views indicate the spatial arrangements of the key residues (His22, Asp121, and Ser25; white) and UDP-glucose (orange) within the sugar donor pockets in the MtUGT71G1 protein. UDP-glucose is abbreviated as UDP-Glc. The hydroxy group of UDP-glucose is shown in the black dotted box. The anomeric carbon of UDP-glucose, which is related to the UGT reaction, is shown with *asterisks*. The side chains of the other amino acid residues forming the sugar donor pockets are shown in green (N-domain residues) or blue (C-domain residues).

**Figure S7.** Virtual docking of UDP-sugars onto homology models of UGT73P12 proteins.

(a) Structural model of the canonical UGT73P12 protein docked with UDP-glucuronic acid.

(b) Structural model of the variant UGT73P12 protein docked with UDP-glucose.

UDP-glucuronic acid and UDP-glucose are abbreviated as UDP-GlcA and UDP-Glc, respectively. The N- and C-domains, and their linker region are visualized in green, blue, and magenta, respectively. The expected positions of the sugar donor pockets in each protein are shown in the orange dotted box.

**Figure S8.** Inhibitory effect of UDP-glucose and UDP-glucuronic acid on the catalytic activity of the canonical UGT73P12 and its R32S mutant protein, respectively (LC-MS chromatogram related to Figure 8d, e).

LC-MS chromatograms of the authentic compounds (glycyrrhetinic acid 3-*O*-monoglucuronide, glycyrrhizin, and glucoglycyrrhizin) and *in vitro* reaction products are shown. The enzyme reactions of the purified proteins (a) canonical UGT73P12 and (b) its R32S mutant protein were individually performed in the presence of glycyrrhetinic acid 3-*O*-monoglucuronide as the sugar acceptor substrate and two competitive sugar donors, UDP-glucuronic acid and UDP-glucose, at various ratios, as described in the EXPERIMENTAL PROCEDURES. A reaction without protein was included as a negative control. UDP-glucuronic acid and UDP-glucose are abbreviated as UDP-GlcA and UDP-Glc, respectively. The LC-MS data represent three independent reactions performed with the same preparation of each purified protein.

**Figure S9.** Enzyme assay of the UGT73B27 and GuUGAT proteins.

(a) SDS-PAGE analysis of purified trigger factor (TF)-tagged recombinant proteins, UGT73B27 and GuUGAT. Site-directed mutagenesis of UGT73B27 to obtain UGT73B27 F329L, which is identical to GuUGAT (Xu *et al.*, 2016), was performed in the entry clone by inverse PCR with the gene-specific primers UGT73B27-F329L-forward (5'-GTGAGAAATTGGAGTGGCTTCCAGAAGGGTTTGAG-3') and UGT73B27-F329L-reverse (5'-CTCAAACCCTTCTGGAAGCCACTCCAATTTCTCAC-3'). The TF protein was used as a negative control. Stain-free gels (Bio-Rad) were used to visualize the UGT bands. The ultraviolet-induced fluorescent signals were detected with the GelDoc XR+ Imaging System (Bio-Rad), and the positions of molecular standards are indicated. *Arrowheads* indicate the UGT bands.

(b) and (c) LC-MS chromatogram of authentic compounds (glycyrrhetinic acid, glycyrrhetinic acid 3-*O*-monoglucuronide, and glycyrrhizin) and *in vitro* reaction products. The enzyme reactions of the purified UGT73B27 and GuUGAT proteins (200 nM) were performed individually for 1 h or 24 h in the presence of glycyrrhetinic acid (10 μM) as a sugar acceptor substrate and UDP-glucuronic acid (50 μM) as a sugar donor using (b) reaction solution 1 containing 50 mM Tris-HCl (pH 7.0), 100 μM MgCl_2_, and 14 mM 2-mercaptoethanol, or (c) solution 2 containing 50 mM Tris-HCl (pH 8.0) and 1 mM DTT (Xu *et al.*, 2016). The reaction with TF protein was used as a negative control. UDP-glucuronic acid is abbreviated as UDP-GlcA. Broken boxes in (b) and (c) indicate that no definite reaction product was detected. The LC-MS data are representative of three separate reactions performed with different preparations of each purified protein.

**Figure S10.** Comparison of structural models of the canonical UGT73P12 and BpUGT94B1 proteins.

The close-up views indicate the predicted spatial arrangements of the key residues (histidine, aspartate, and arginine; N-domain residues; white) and UDP-glucuronic acid (orange) within the sugar donor pockets in each protein. UDP-glucuronic acid is abbreviated as UDP-GlcA. Possible electrostatic interactions among the key residues and UDP-glucuronic acid are shown with black dotted lines. The anomeric carbon of UDP-glucuronic acid, which is related to the UGT reaction, is shown with *asterisks*. The side chains of the other amino acid residues forming the sugar donor pockets are shown in green (N-domain residues), blue (C-domain residues), or magenta (linker residues).

**Figure S11.** Summary of the catalytic functions of the UGT73P12 and UGT73P13 proteins.

**SUPPLEMENTARY REFERENCES**

**Kubo, A., Arai, Y., Nagashima, S. and Yoshikawa, T.** (2004) Alteration of sugar donor specificities of plant glycosyltransferases by a single point mutation. *Arch. Biochem. Biophys.*, **429**, 198-203. https://doi.org/10.1016/j.abb.2004.06.021

**Noguchi, A., Horikawa, M., Fukui, Y., Fukuchi-Mizutani, M., Iuchi-Okada, A., Ishiguro, M., Kiso, Y., Nakayama, T. and Ono, E.** (2009) Local differentiation of sugar donor specificity of flavonoid glycosyltransferase in Lamiales. *Plant Cell*, **21**, 1556-1572. https://doi.org/10.1105/tpc.108.063826

**Sayama, T., Ono, E., Takagi, K., Takada, Y., Horikawa, M., Nakamoto, Y., Hirose, A., Sasama, H., Ohashi, M., Hasegawa, H., Terakawa, T., Kikuchi, A., Kato, S., Tatsuzaki, N., Tsukamoto, C. and Ishimoto, M.** (2012) The *Sg-1* glycosyltransferase locus regulates structural diversity of triterpenoid saponins of soybean. *Plant Cell*, **24**, 2123-2138. https://doi.org/10.1105/tpc.111.095174

**Xu, G., Cai, W., Gao, W. and Liu, C.** (2016) A novel glucuronosyltransferase has an unprecedented ability to catalyse continuous two-step glucuronosylation of glycyrrhetinic acid to yield glycyrrhizin. *New Phytol.*, **212**, 123-135. https://doi.org/10.1111/nph.14039
